# Recent advances in carbon quantum dots for virus detection, as well as inhibition and treatment of viral infection

**DOI:** 10.1186/s40580-022-00307-9

**Published:** 2022-04-02

**Authors:** Yuxiang Xue, Chenchen Liu, Gavin Andrews, Jinyan Wang, Yi Ge

**Affiliations:** 1 Institute for Bioengineering, School of Engineering, University of Edinburgh, Edinburgh, EH9 3HL UK; 2 Department of Metabolism, Digestion and Reproductive, Faculty of Medicine, Imperial College London, London, SW7 2AZ UK; 3 School of Pharmacy, Queen’s University Belfast, Belfast, BT9 7BL UK; 4 College of Basic Medical Science, China Medical University, Shenyang, 110122 China

**Keywords:** Carbon quantum dot, Carbon dot, Nanomaterial, Virus detection, Antiviral agent, SARS-CoV-2, COVID-19

## Abstract

In the last decade, carbon quantum dots (CQDs), as a novel class of carbon-based nanomaterials, have received increasing attention due to their distinct properties. CQDs are ultimately small nanoparticles with an average size below 10 nm, possessing high water solubility, alluring photoluminescence, photostability, excellent biocompatibility, low/none toxicity, environmental friendliness, and high sustainability, etc. In history, there are intermittent threats from viruses to humans, animals and plants worldwide, resulting in enormous crises and impacts on our life, environment, economy and society. Some recent studies have unveiled that certain types of CQDs exhibited high and potent antiviral activities against various viruses such as human coronavirus, arterivirus, norovirus and herpesvirus. Moreover, they have been successfully explored and developed for different virus detections including severe acute respiratory syndrome coronavirus 2 (SARS-CoV-2). This article exclusively overviews and discusses the recent progress of designing, synthesizing, modifying/functionalizing and developing CQDs towards effective virus detection as well as the inhibition and treatment of viral infection. Their mechanisms and applications against various pathogenic viruses are addressed. The latest outcomes for combating the coronavirus disease 2019 (COVID-19) utilizing CQDs are also highlighted. It can be envisaged that CQDs could further benefit the development of virus detectors and antiviral agents with added broad-spectrum activity and cost-effective production.

## Introduction

Humans have been fighting viruses throughout history, from plague, variola virus, swine flu virus, Ebola, and HIV (human immunodeficiency virus) to SARS (severe acute respiratory syndrome), MERS (middle east respiratory syndrome coronavirus) and Zika, which have costed billions of lives and made severe socio-economic impacts irreversibly. In the end of 2019, a new coronavirus, namely SARS-CoV-2 (severe acute respiratory syndrome coronavirus 2), was first reported and then rapidly spread over the world. Nowadays, the coronavirus disease 2019 (COVID-19), caused by SARS-CoV-2, has become a major and ongoing threat to human health. The COVID-19 pandemic is considered as one of the deadliest pandemics in human history. Amid this unprecedented global crisis, it has now never been more imperative and urgent to exploit, design and develop effective antiviral agents and diagnostic methods. Table 1 summaries some major life-threatening viruses, together with their main treatment and diagnostic methods.

**Table 1:** Some major life-threatening viruses and examples of their main treatment and diagnostic methods

Types of virus	Caused disease	Example of treatment method	Example of diagnostic method	Refs.
Variola virus	Smallpox	Vaccines and antiviral agents/drugs (e.g*.* tecovirimat, cidofovir and brincidofovir)	Immune serum complement fixation test; physical exam of skin	[1–3]
HIV	Acquired immunodeficiency syndrome (AIDS)	Antiretroviral agents/drugs (e.g. NRTIs and NNRTIs) HIV protease inhibitors (e.g. mozenavir) HIV vaccines (e.g. neutralizing antibodies and recombinant viral vectors)	PCR or viral load test; P24 test; ELISA; rapid finger prick and oral swab test; Western blot test	[4]
SARS	Severe acute respiratory syndrome (SARS)	Antiviral agents/drugs (e.g*.* ribavirin, corticosteroid, and type 1 IFN); convalescent plasma and immunoglobulin	Serology; real time reverse transcription PCR; ELISA	[5]
MERS	Middle east respiratory syndrome (MERS)	Antiviral agents/drugs (e.g. type 1 IFN, IFN-α2b combined with ribavirin, chloroquine, and lopinavir); convalescent sera from recovered patients	Serology; real time reverse transcription PCR; ELISA; Immunofluorescence assay	[6]
Ebola	Ebola hemorrhagic fever	Monoclonal antibodies (e.g. REGN-EB3, mAb114, and ZMapp); antiviral agents/drugs (e.g*.* remdesivir)	Quantitative reverse transcription PCR; ELISA antigen capture; Ebola-specific IgM and IgG antibody detection	[7]
SARS-CoV-2	Coronavirus disease 2019 (COVID-19)	Vaccines (e.g. inactivated vaccines, mRNA vaccines, recombinant protein vaccines, and live attenuated vaccines); antiviral agents/drugs (e.g. umifenovir; lopinavir, ritonavir, tocilizumab, and sarilumab)	Real time reverse transcription PCR by using nasal swab or sputum sample; blood and antibody test	[8, 9]

Nanotechnology has the potential of producing new materials and products that may revolutionize all areas of life. It has already been recognized as one of the six key enabling technologies that will transform our society in the foreseeable future [10]. Nanomedicine now plays a pivotal role in healthcare and quality of life for patients, greatly redefining many biomedical and pharmaceutical fields such as imaging, diagnosis, therapeutics, drug delivery, and formulation [11].

Various nanoscale materials have emerged as novel antiviral agents owning to their unique physical and chemical properties [12–14]. They have also been broadly applied in diagnosis of viral infections.

In the last decade, carbon quantum dots (CQDs), as a new class of nanomaterials, have received increasing attention due to their distinct characters. CQDs are ultimately small particles with an average size smaller than 10 nm, possessing high water solubility, alluring photoluminescence, photostability, excellent biocompatibility, low/none toxicity, environmental friendliness, and high sustainability, etc. [15–17]. Recently, some studies have unveiled that certain types of CQDs have exhibited high and potent antiviral activities against human coronavirus, arterivirus, norovirus and herpesvirus [18–22]. More interestingly, a benzoxazine monomer derived CQDs was recently reported as a broad-spectrum agent to block viral infectivity against life-threatening flaviviruses (Japanese encephalitis, Zika, and dengue viruses) and non-enveloped viruses (porcine parvovirus and adenovirus-associated virus) [23].

**Fig. 1:**
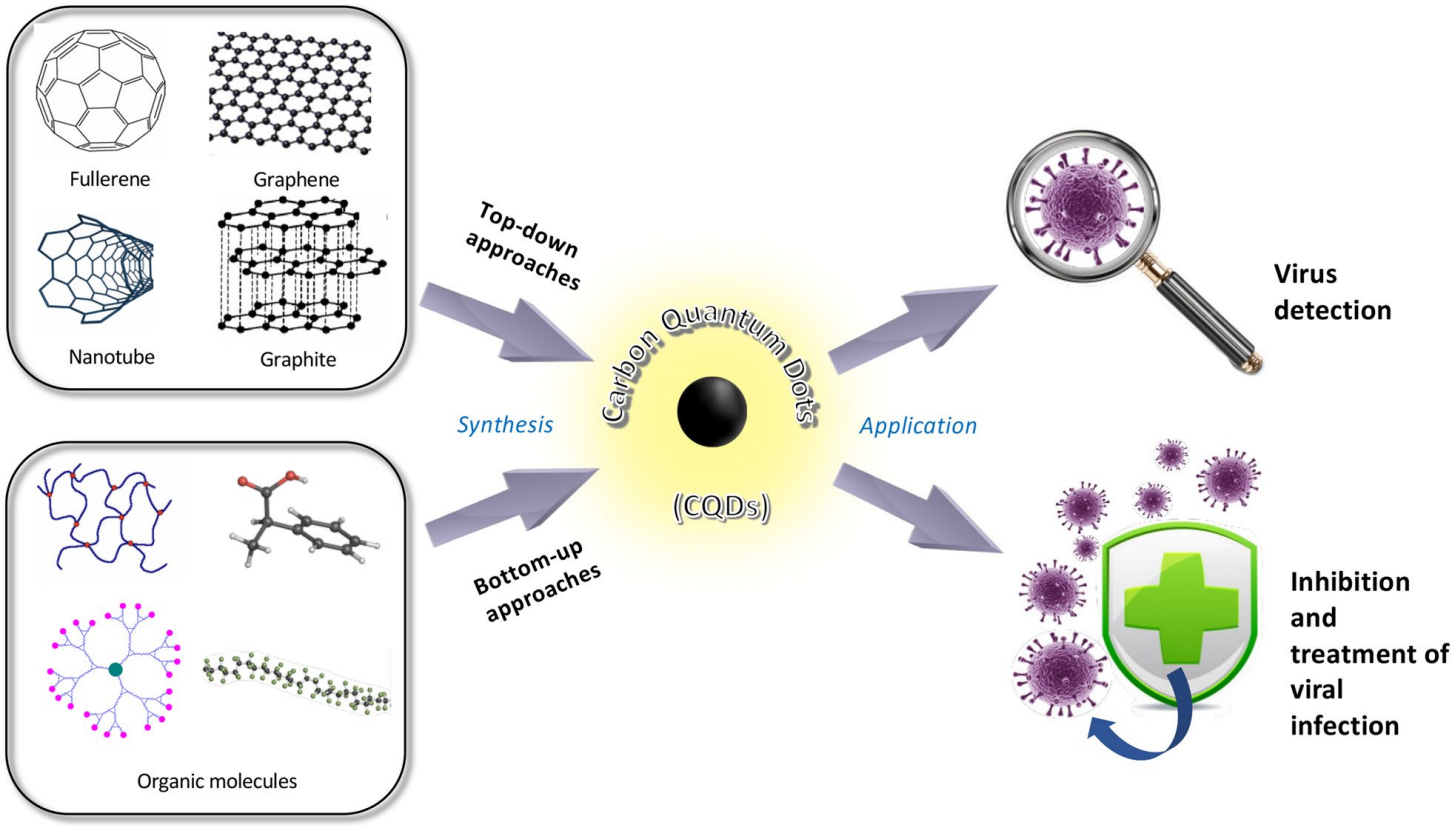
Schematic representation of synthesizing carbon quantum dots for the detection, prevention/inhibition, and treatment of pathogenic viruses

In this article, we provide a comprehensive and up-to-date overview on the structure, type, synthesis, property and biomedical application of CQDs, particularly with a focal point on their mechanisms and roles/applications for the detection, prevention/inhibition, and treatment of various pathogenic viruses (Fig. 1). The related studies on combating COVID-19 utilizing CQDs are also timely addressed and discussed.

## Viruses and nanomaterials

### General viral pathogenesis

Viruses themselves have evolved and developed many different pathogenic mechanisms to cause diseases. In general, these pathogenic mechanisms include the entry, replication and spread of virus inside the body, the tissue damage development as well as immune response [24]. A better understanding of viral pathogenesis could efficiently facilitate the design of antiviral agents, virus detection and prevention of viral infection, etc.

**Fig. 2:**
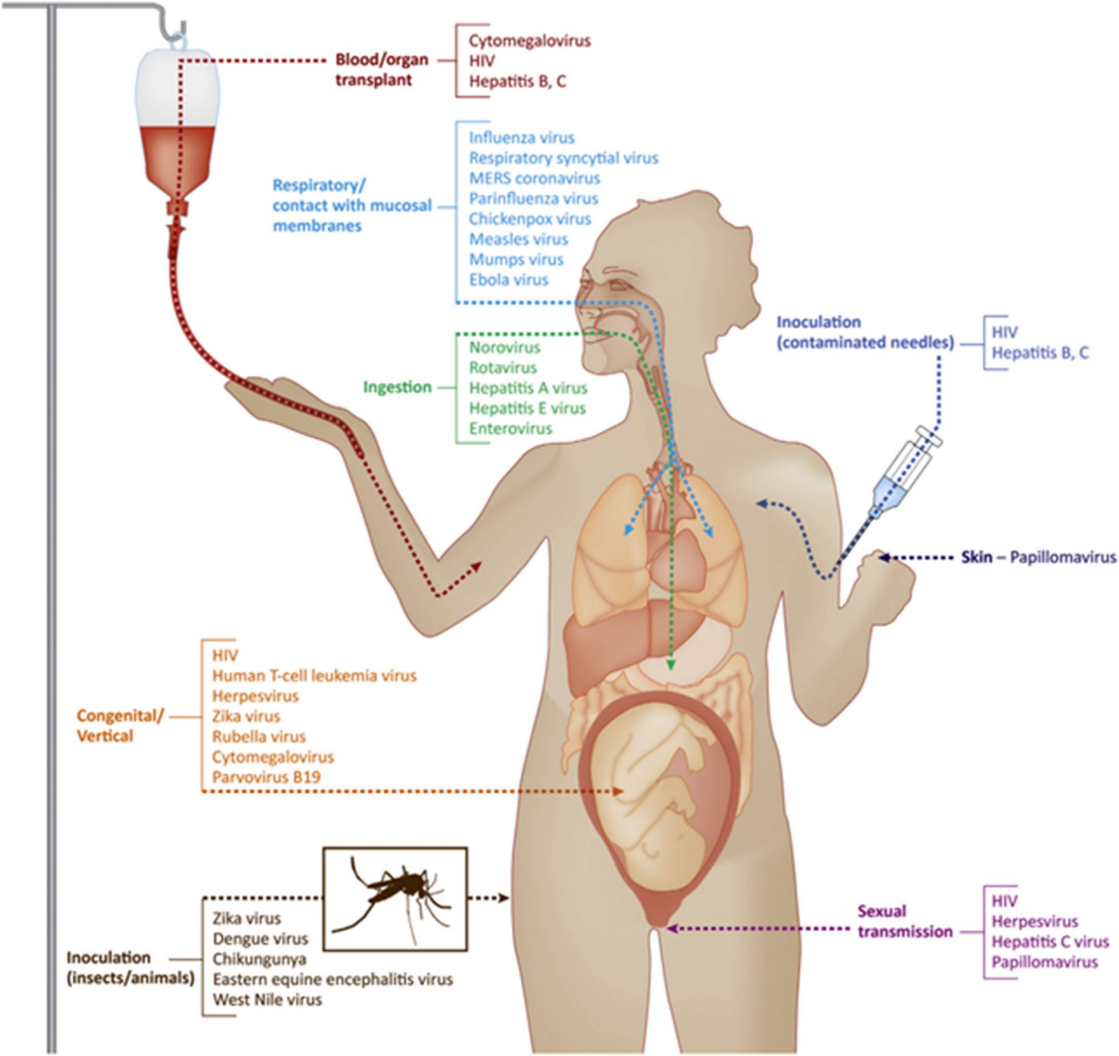
General entry routes of viruses [27]. Reproduced with permission. Copyright © 2017, Elsevier B.V

For various pathogenic viruses, there are different routes of entry (Fig. 2) such as entry via the respiratory tract which is one of the most popular and primary entry sites of viruses into the body [25]. Viruses entering the body via the respiratory tract could subsequently bind to specific receptors on epithelial cells. For example, SARS-CoV-2 and SARS are able to bind angiotensin converting enzyme 2 (ACE2) [26]. As a comparison, some other viruses could enter the body via skin (e.g. papillomaviruses), genitourinary tract (e.g. herpes simplex virus 2 and HIV), or alimentary tract (e.g. rotaviruses and noroviruses).

After the entry, the replicated pathogenic viruses may remain localized and/or become systemic/generalized via lymphatic and hematogenous routes. Viruses such as SARS-CoV-2, SARS or MERS that enter the body via the respiratory tract can spread quickly through the layer of fluid/mucus that covers epithelial surfaces, thus the infections caused by these viruses often progress and deteriorate rapidly [28].

Different viruses have different distinct patterns of infection, which are mainly based on the differences in their major sites (tissues/organs) of replication and damage. For instance, SARS-CoV-2 could actively replicate in the upper respiratory tissues as well as lower respiratory organs since it targets ACE2 as the main receptor, which is mainly expressed in respiratory endothelium, alveolar monocytes and macrophages [29].

An individual who has a viral infection may or may not display disease symptom(s). Only when the damages are induced by the virus or host immune system, disease is resulted. For example, SARS-CoV-2 infected cells can cause the excessive production of pro-inflammatory cytokines, which damage the tissue of lungs and affect the gas exchange function of lung [30]. The published single-cell RNA sequencing data have shown that transmembrane protease serine protease 2 (TMPRSS2) is highly expressed in nasal epithelial cells, lungs and bronchial branches and is co-expressed with ACE2, which explains the tissue specificity of SARS-CoV-2 [31]. After binding with respiratory epithelial cells ACE2, SARS-CoV-2 begins to replicate rapidly and migrate down to the respiratory tract and entry the pulmonary alveolar epithelial cells. The rapid replication of SARS-CoV-2 in the lung triggers a strong proinflammatory cytokine response, which causes inflammatory cell infiltration and then damage the lung tissues [32]. Moreover, according to damage response framework (DRF), when the host damage reaches the threshold of damaging homeostasis, clinical symptoms will appear [33]. In COVID-19 caused by SARS-CoV-2, the clinical symptoms range from mild upper respiratory disease to life-threatening acute respiratory syndrome. In the latter, oxygenation is affected by lung inflammation, which is also a reflection of host damage caused by immune system attack [34].

### Detection of virus utilizing nanoparticles

Since lots of pathogenic viruses are lack of effective drugs for clinical treatment, a rapid and accurate detection of virus in the early stage has become crucial for preventing the spread of pathogens. The existing methods including serological antibody test or reverse transcription PCR however are yet sensitive and accurate enough to detect viruses in clinical samples [35].

Nanomaterials have shown a variety of unique optical, electrical, magnetic and mechanical properties, and they have been widely applied in sensing and diagnosis [36, 37]. For example, metal and metal oxide nanoparticles, such as gold NPs (AuNPs), silver NPs (AgNPs), aluminum NPs (AlNPs) and iron oxide NPs have been successfully developed for virus detection [38]. The nano-biological hybrid systems that contain and bind one or more biomolecules (e.g. DNA, RNA, antibodies, antigens and peptides) derived from viruses and metal NPs, are commonly assembled and used. In such a way, a simple, fast, highly sensitive, label-free and/or multiplex detection method could be achieved owing to the intrinsic and advanced properties of metal NPs, such as high surface area, good stability, biocompatibility, high degree of surface functionalization and tunable physico-chemical properties. Recently, various efforts have been paid by using AuNPs as biosensors to detect HBV (hepatitis B virus), using MoS_2_@Cu_2_O-Pt nanohybrids as enzyme-mimetic label for the detection of hepatitis B surface antigen, and using gold-coated iron oxide nanoparticle HBV DNA probes for HBV diagnosis [39–41].

Apart from metal and magnetic nanoparticles, other nanomaterials such as carbon nanotubes (CNTs) and silica nanoparticles (SiNPs) have also been applied for virus detection. Some researchers designed and formulated a NiCo-based metal–organic framework (i.e. NiCo_2_O_4_/CoO@CNTs) to detect HIV-1, and showed that this system had high electrochemical activity, biocompatibility, and strong bio-affinity toward the probe DNA [42]. Besides, this system also could detect human serum samples with good stability and reproducibility. Chunduri et al*.* reported that the streptavidin labelled and Eu-doped fluorescent SiNPs could be applied for HIV-1 p24 antigen detection with high specificity which is about 1000-fold enhancement over conventional colorimetric ELISA [43]. The information of various nanoparticles used for viral detection is summarized in Table 2.

**Table 2:** Various nanoparticles used for viral detection

Type of nanoparticle	Application	Principle	Refs.
AuNPs	Detection of human papillomavirus in cervical carcinoma	AuNPs were coupled with silver straining for signal amplification	[44]
	Detection of surface antigen of HBV in biological sample	AuNPs were surface labelled with a monoclonal HBV surface antibody and the technique was validated by ELISA	[45]
	Detection of HCV RNA	Using size- and distance-dependent nanoparticle surface-energy transfer technique. When RNA bound to AuNPs, the color of solution was changed from red to yellow	[46]
	Detection of HVA Vall7 polyprotein gene, HVB surface antigen gene, HIV, Ebola virus, variola virus (smallpox), and Bacillus anthracis (BA) protective antigen gene	AuNPs were probes labeled with oligonucleotides and Raman-active dyes to achieve surface-enhanced Raman scattering	[47]
	Detection of SARS Detection of SARS-CoV-2	Mainly through colorimetry (pp1ab gene detection) and electrochemical methods (nucleocapsid protein gene detection) for rapid and specific molecular detection A colorimetric assay where the thiol-modified antisense oligonucleotides-capped AuNPs accumulated selectively in the presence of its target RNA sequence in SARS-CoV-2 and showed the plasmon resonance change on the surface	[48] [49]
	Detection of Hantaan virus nucleocapsid protein	Using functionalized AuNPs to enhance the ultrasensitive immuno-PCR assay based on bio-barcode assay technique	[50]
	Detection of Ebola virus	Using a luminescence assay consisting of BaGdF5:Yb/Er up-conversion nanoparticles conjugated with oligonucleotide probe and AuNPs linked with target Ebola virus oligonucleotide	[51]
AgNPs	Detection of single influenza viruses	Using the inherent electrochemical activity of virus surface modified by AgNPs in the solution to detect virus at low concentration quantitatively	[52]
	Early detection of COVID-19	Redox probes containing the silver ions (Ag^+^) in the hexathia-18-crown-6 (HT18C6) were used for voltametric determination of RdRP of SARS-CoV-2 virus	[53]
AlNPs	Detection of dengue virus	A small and thin piece of nano-porous alumina membrane was used to detect virus using electrochemical impedance spectroscopy	[54]
Iron oxide NPs	Detection of hepatitis A virus (HAV)	Using protamine-coated iron oxide (Fe_3_O_4_) magnetic nanoparticles to concentrate HAV for further detection	[55]
	Detection of hepatitis B virus (HBV)	Using gold-coated iron oxide nanoparticle as the HBV DNA probe	[41]
Magnetic NPs	Detection of infuenza A virus H5N1	Aniline monomer polymerized around gamma iron (III) oxide (γ-Fe_2_O_3_) cores were served as the basis of a direct-charge transfer biosensor to detect surface glycoprotein hemagglutinin of influenza A virus	[56]
	Detection of hepatitis B virus (HBV)	Amino functionalized carbon coated magnetic nanoparticles were used for electrochemical detection of hybridization of nucleic acid of HBV	[57]
CNTs	Detection of HIV-1	Using NiCo2O4/CoO@CNTs which have high electrochemical activity, good biocompatibility, and strong bio-affinity toward the probe DNA	[42]
	Detection of SARS-CoV-2	Target SARS-CoV-2 viral RNA was captured by ssDNA-nanotube constructs via hybridization and separated from the liquid phase in a single-tube system with minimal chemical reagents, for downstream quantitative reverse transcription PCR detection	[58]
SiNPs	Detection of HIV-1 Detection of Hepatitis B virus (HBV)	Using Streptavidin-labelled and Europium-doped fluorescent SiNPs for HIV-1 p24 antigen detection Using Fe_3_O_4_/SiO_2_ nanoparticles to isolate genomic DNA of HBV for HBV detection based on PCR	[43] [59]
Polymeric NPs	Detection of airborne respiratory viruses	TMB-NPs@PLGA-based colorimetric sensor was conjugated to antibodies and bound to the captured virus in the microtiter wells	[60]
Graphenes	Detection of HIV	Amine-functionalized graphene was conjugated with anti-p24 of HIV to detect various HIV biomarkers via various UV–Vis and Raman spectroscopies	[61]

### Nanoparticles as antiviral agents

Currently, the existing conventional antiviral agents/drugs are still facing many drawbacks and challenges such as drug resistance, side effect, narrow spectrum, and high cost [62, 63]. Advanced nanomaterials, which are capable of entering cells and inhibiting virus replication, have been successfully developed as not only drug carriers for antiviral agents but also antiviral agents themselves [64]. Some recent studies have revealed that certain nanoparticles as antiviral agents could effectively reduce the risk of drug resistance, and give a broad-spectrum performance [38, 65]. Some other functional nanoparticles are able to attack and kill viruses photothermally or via photocatalysis-induced reactive oxygen species (ROS) [66]. In general, various metal, metal oxide and hybrid nanoparticles have been successfully developed and applied as antiviral agents (Table 3).

**Table 3:** Various nanoparticles which have been applied as antiviral agents

Type of nanoparticle	Application	Principle	Refs.
Iron oxide NPs	Inactivation of influenza	Using iron oxide nanozymes to catalyze lipid peroxidation of the viral lipid envelope to inactivate enveloped viruses	[67]
	Inactivation of SARS-CoV-2	IONPs (e.g. Fe_2_O_3_ and Fe_3_O_4_) could interact with the spike protein receptor binding domain (S1-RBD) of SARS-CoV-2 that is required for virus attachment to the host cell receptors	[68]
ZnO-NPs	Inhibition of H1N1 influenza virus	PEGylated ZnO-NPs could inhibit H1N1 influenza by blocking viral entry	[69]
AuNPs	Inhibition of HIV infection	AuNPs were coated with multiple copies of an amphiphilic sulfate-ended ligand which could bind the HIV gp120	[70]
Ag NPs	Inhibition of growth of H3N2 influenza virus	AgNPs could interact with H3N2 influenza virus and lead to the destruction of morphologic viral structures	[71]
	Inhibition of HIV replication	Blocking of viral entry and having an interference with viral membrane fusion in a short period of time	[12]
	Inhibition of H1N1 influenza A virus	AgNPs were combined with chitosan to inhibit viral penetration into the host cell by direct binding with viral envelope glycoproteins	[72]
	Inhibition of herpes simplex virus type 1 (HSV-1)	AgNPs were capped with mercaptoethane sulfonate (Ag-MES) inhibiting HSV-1 to have the interaction between viral envelope glycoproteins and cell surface heparan sulfate	[73]
	Inhibition of hepatitis B virus replication	AgNPs which have good binding affinity for HBV DNA inhibited the in vitro production of HBV RNA and extracellular virion	[74]
Hybrid nanocomposites	Inhibition of enveloped virus	GO sheets were combined with AgNPs to inhibit the infection of viruses with low cytotoxicity to cells	[75]
	Inhibition of herpes simplex virus type 1 (HSV-1)	Sulfonated magnetic nanoparticles were functionalized with reduced graphene oxide showing a photothermal antiviral activity	[76]

## Carbon quantum dots

**Fig. 3:**
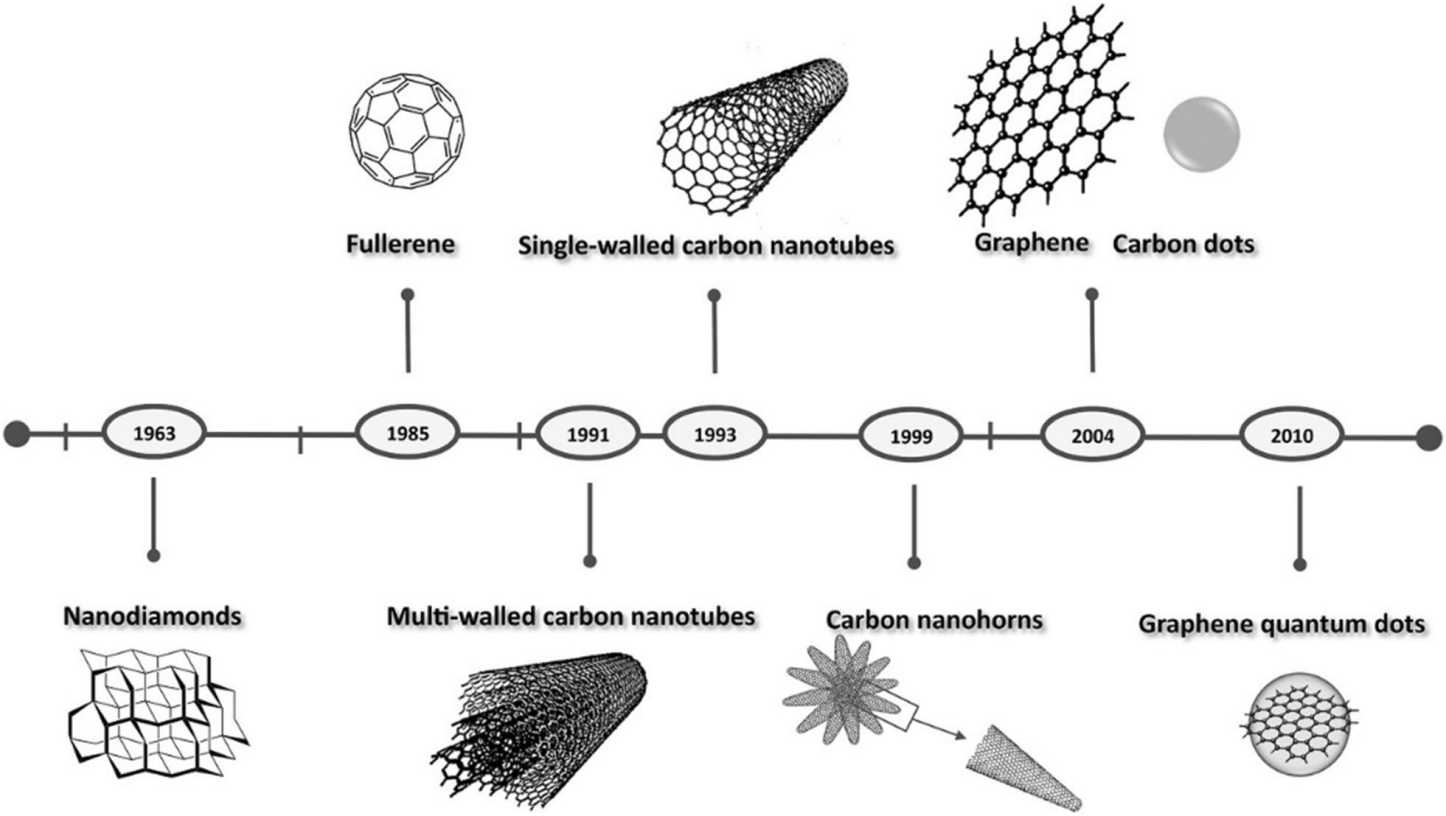
The major milestones in the development of carbon-based nanomaterials [85]. Reproduced with permission. Copyright © 2020 WILEY‐VCH Verlag GmbH & Co. KGaA, Weinheim

Carbon quantum dots (CQDs), also known as carbon dots (CDs), are new kind of fluorescent subclass in the category of carbon-based nanomaterials (Fig. 3) with an average size usually smaller than 10 nm [77]. CQDs were first reported in 2004 by Xu et al. in a research on single-walled carbon nanotube fragments [78]. In their experiments, a mixture of whole new fluorescent nanoparticles were separated during purification of single-walled carbon nanotubes. Since then, different CQDs have been prepared with various methods [79, 80]. Great progresses in many aspects, such as derivatization/modification and functionalization, have further been made [81, 82].

Due to some of their advanced properties such as distinct optical properties and conductivity, high chemical stability, great biocompatibility, low-cost and ease of modification, CQDs have shown tremendous capability and potential for various applications especially biomedical applications [83, 84]. CQDs can also be made from many natural materials, which endow them with some unique features such as green preparation and cost-effectiveness.

### Structure and type of carbon quantum dots

Although there are some controversies concerning the basic theory of CQDs, it is generally accepted that CQDs are zero-dimensional nanomaterials composed of a carbon skeleton and surface functional groups [86]. The particle size of CQDs is generally in the range of 2–10 nm [87]. The internal structure is composed of sp^2^ and sp^3^ hybridized carbon atoms while the outer structure is composed of sp^3^ hybridized carbon atoms [88, 89]. In light of their chemical structures, the CQDs covers a relative wide range of forms. Although there have no unified classifying standards of CQDs in the literature, according to their specific carbon core structures and morphologies, CDs can be divided into CQDs, graphitic structure CQDs, carbonized polymer dots (CPDs) and CQDs with a C_3_N_4_ crystalline core. Among them, spherical and single-sheet carbon-based nanoparticles CQDs are the most reported ones [90]. The most distinctive feature of CQDs is their size dependent photoluminescence. Compared with graphite, the X-ray diffraction (XRD) pattern of CQDs appear two characteristic peaks at 22.59° and 18.20° indicating the existence of amorphous carbon and hexagonal carbon [91]. Multiple studies have shown that under certain conditions, nitrogen atom can dope into the carbon core of CQDs forming special nitride structures (g-C_3_N_4_ or β-C_3_N_4_) [92, 93]. When the nitrogen doping agent in starting material reached a certain threshold, the core structure of CQDs was changed and the carbon nitride nanocrystals were resulted [94]. Those CQDs with a C_3_N_4_ crystalline core usually exhibit good optical properties and photocatalytic activity [95].

Graphene quantum dots (GQDs) are kinds of carbon nanomaterials family which contains π-conjugated single sheets [96, 97]. They have a graphene lattices structure and contain one or more layers of graphene sheets which have thickness less than 5 nm [98, 99]. Compared with conventional CQDs, GQDs have more crystalline sp^2^ carbon atoms and less crystal defects. In the Raman spectrum, GQDs show a similar D and G bands with graphene but higher *I*_*D*_*/I*_*G*_ ratios due to higher proportion of sp^3^-hybridised carbons at the edges. Because of the small dimension of GQDs, quantum confinement and edge effects become more predominant [100]. Thus GQDs have nonzero band gaps while large graphene nanosheets usually exhibit a band gap of zero width [86]. This feature equips GQDs with excellent electronic and optical properties as other CQDs [100].

CPDs are a new concept that has been proposed to describe those CQDs which possess highly dehydrated crosslinking polymer frames [101]. During the synthesis reaction, the intermediate undergo a complex process including polymerization, dehydration and carbonization and finally obtain polymer/carbon hybrid structures [102]. Therefore, CPDs are sometimes regarded as a special type of CQDs. By controlling carbonization, CPDs can be transferred into CQDs [101]. One of the main differences between CQDs and CPDs is photoluminescence mechanism. The optical properties of CPDs are mainly dominated by cross-link-enhanced emission [103]. CPDs contain a large number of sub-fluorophore groups such as heteroatom-containing double bonds and single bonds. These sub-fluorophore groups have weak potential photoluminescence properties due to intramolecular rotation and vibration. In CPDs, due to chemical crosslinking or physical aggregation, intramolecular rotation and vibration were restricted and therefore an enhanced photoluminescence property can be observed [104, 105].

There is a controversy concerning whether CQDs possess a general crystal structure. In the initial studies, the reported CQDs usually possessed the amorphous characteristic which exhibited a broad diffraction pattern under XRD [106–108]. However, by selecting appropriate synthetic route and starting material, CQDs could exhibit crystalline nature. In recent years, most reported CQDs have a crystalline structure [109]. The crystal core of CQDs can be monocrystalline or polycrystalline [93, 109]. It is worth noting that the optical properties of CQDs could be affected by their crystal structures. Typically, CQDs contain a sp^3^-hybridised amorphous carbon core. However, sp^2^-hybridized crystalline domains can be generated under specific conditions. In sp^3^-hybridized carbon atoms, the valency electrons are localized at stable σ-bonds and are only sensitive to high-energy UV light while in sp^2^-hybridized carbon atoms. The valency electrons are delocalized over the entire domain area, thus having a wide absorption in the visible spectral range [110]. Through theoretical calculation, Tepliakov et al. found that the experimental optical properties of CQDs were highly dependent on sp^2^-hybridized atomic domains. The absorption spectrum could be changed by altering the distribution of the hybridization domains [110]. Besides, compared with crystalline CQDs, amorphous CQDs are more susceptible to Photobleaching, indicating the photostability has close touch with arrangement structure of CQDs [111].

### Synthesis of carbon quantum dots

**Fig. 4:**
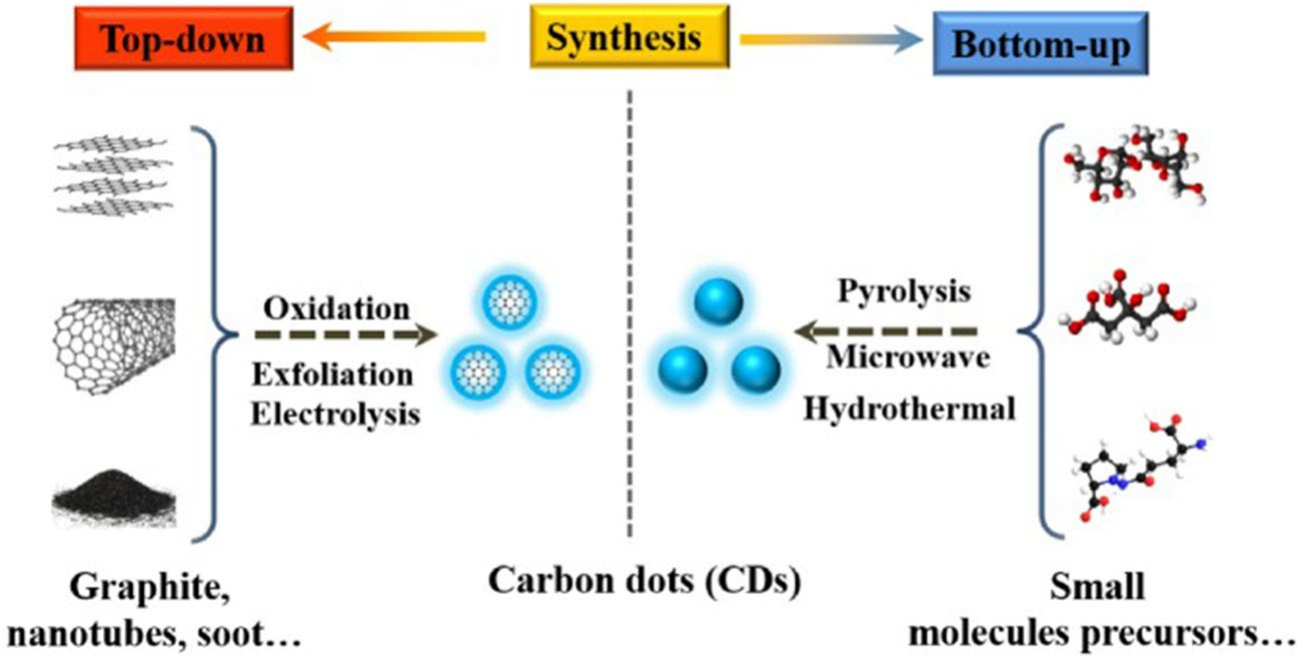
A schematic representation of general synthetic approaches of carbon quantum dots [112]. Reproduced with permission. Copyright © 2022 Wiley‐VCH GmbH

To date, a wide variety of synthetic pathways have been reported to harvest CQDs with different physical and chemical properties. They can further easily be optimized by adjusting reaction conditions and reactants. According to the carbon sourced used in synthesis, the synthetic pathways of CQDs can generally be categorized into “top-down” and “bottom-up” approaches (Fig. 4) [112].

The “top-down” approach is usually a physical or chemical method, which produces small-sized CQDs by peeling from a large-scale carbon source such as activated carbon, carbon fiber, carbon nanotubes and graphite rod. The “bottom-up” method always starts from small molecule carbon sources such as glucose, critic acid, folic acid, etc. Table 4 summarize some recently reported synthetic CQDs based on these two approaches applying different carbon source, synthetic method with varied quantum yield and application.

**Table 4:** Comparison of some recently reported synthetic CQDs

Approach	Carbon source	Synthetic method	Quantum yield (%)	Application	Refs.
Top-down	Carbon nano powder	Acidic oxidation	N/A	Drug delivery	[113]
Top-down	Graphite flakes	laser ablation	9.1	N/A	[114]
Top-down	Graphite rod	Electrochemical exfoliation, acidic oxidation	15.5	Bioimaging	[115]
Top-down	Graphite	Arc discharge	N/A	Emitting materials	[116]
Top-down	Graphite powder	Acidic oxidation	14	N/A	[117]
Bottom-up	Citric acid	Hydrothermal	51	Alcohol sensors	[118]
Bottom-up	Glycyrrhizic acid	Hydrothermal	1.41	Anti-virus	[20]
Bottom-up	L-cysteine and citric acid	Microwave irradiation	54	Glutathione detection	[119]
Bottom-up	Gallic acid	Microwave irradiation	25	Bioimaging, Anti-tumor	[120]
Bottom-up	Oligomer polyamide resin	Ultrasonic treatment	28.3	Photoluminescent ink	[121]
Bottom-up	Toluene	Laser irradiation	13.5	N/A	[122]
Bottom-up	o-phenylenediamine	Electrochemical method	71	Bioimaging	[123]

#### “Top-down” approaches

Usually, fabricating CQDs by cutting higher dimensional bulk carbon precursors is recognized as the “top-down” approach. This approach includes laser ablation, electrochemical carbonization, chemical ablation, and hydrothermal/solvothermal/special oxidation cleavage, etc [85, 124]. In this approach, inexpensive raw carbonaceous materials are usually used as precursors. Due to the relatively simple synthetic procedures, CQDs can also be easily manufactured in a large scale [125].

The first top-down synthesis of CQDs was reported by Xu et al*.* through arc discharge and it is also the first reported work for a successful synthesis of CQDs [78]. However, the CQDs derived from arc discharge method always face the problem of low yield, complex compositional heterogeneity and are hard to be purified [126]. By contrast, CQDs are much easier to be purified through electrochemical carbonization. In the traditional electrochemical method, CQDs are peeled directly from graphite electrodes under the action of redox reaction induced by an electric field in the electrochemical cell [124]. Such an approach further allows to synthesis CQDs cheaply and easily. By adjusting some reaction conditions such as electrolyte concentration and current intensity, CQDs with different physical and chemical properties can be obtained. Tan and his co-workers successfully synthesized red fluorescent GQDs by using an electrochemical method to exfoliate graphite in K_2_S_2_O_8_ electrolyte solution [127]. When the K_2_S_2_O_8_ electrolyte solution was replaced to K_2_FeO_4_ electrolyte solution, no GQDs were formed while only a small amount of GQDs were observed when electrolyte solution was replaced to Na_2_SO_4_ solution. It was suggested that the generated sulfate radicals in the electrochemical reaction acted as scissors cutting the graphene sheets into GQDs. In another study, Devi et al*.* observed that the particle size of CQDs is affected by the changes of applied current [128]. In addition, in recent years it has been reported that small molecular organic matter can also be used as precursor to synthesize CQDs via electrochemical methods. For example, Deng et al*.* fabricated CQDs by using a platinum electrode to electrolyze low-molecular-weight alcohol solution [129]. As a result, the CQDs were be prepared by using ethanol as carbon source through carbonization and dehydration in alkaline solution.

As another relatively early method applied for the synthesis of CQDs, the laser ablation method was first used by Sun et al*.* in 2006 to etch carbon target in mixed gases [130]. There are generally two main laser ablation-based methods to synthesis CQDs. One method is to use laser to etch solid carbon source (e.g. glassy carbon plate) which is immersed in water, while the other method is to etch carbon powder (e.g. graphite powder) which is suspended in water [131, 132]. By employing the laser ablation method, the carbon skeleton is destructed by photothermal vaporization and coulomb explosion in the strong laser field. As a result, the large carbon source breaks up into small CQDs. The laser ablation method is regarded as a green and simple method and the purity of product is relatively high. However, during the fabrication process, the solution tends to become turbid leading to loss of laser energy and thus impacts the properties of CDs. Besides, since the liquid volume is too large compared to the radiation volume, the reaction is always incomplete and hence it is necessary to remove unreacted materials afterwards.

Chemical oxidation is sometimes called Hummers’ method, which uses strong oxidants (i.e. nitric acid, sulfuric acid and hydrogen peroxide) to treat carbon precursor at high temperature for CQDs synthesis [98]. Under normal circumstances, the carbon source could be a macromolecular material (e.g. graphene) or a small molecule substance (e.g. sucrose) [133]. This method possesses merits of easy operation, high reproducibility and short reaction time [134]. In addition, the CQDs produced by this method usually exhibited excellent fluorescence property [135, 136]. Souza et al*.* recently developed a low cost synthesis pathway for the fabrication of fluorescent CQDs by using bleached eucalyptus kraft pulp as the raw material [137]. The resulting CQDs had an average particle size of 2 nm. The large number of carboxylic acid group on the surface of these CQDs provided sufficient functional groups for a further modification. After the modification with PEG molecules, their quantum yield (QY) was increased from 1.2% to 3.2%. In this method, carbonaceous material was oxidized by concentrated sulfuric acid after the dehydration and carbonization of organic precursor, and then broken down into small carbons. During this process, various oxygenated functional groups such as carboxylic group, hydroxyl group and carbonyl group could be introduced to the resulting CQDs.

#### “Bottom-up” approaches

The method used for synthesizing CQDs from small molecular carbon precursors could be classified as the “bottom-up” approach. It generally includes hydrothermal method, microwave irradiation method and ultrasonic oscillation method, etc. [134, 138] In comparison with the “top-down” approach, CQDs derived from the “bottom-up” approach always exhibited high QY and strong photoluminescence intensity [139]. More importantly, through the use of various precursors, the chemical structure and physical properties of synthesized CQDs can be readily tuned. In recent years, this approach has been increasingly applied more than the “top-down” approach.

The microwave-assisted irradiation method has been widely used in rapid and green synthesis of CQDs which have high fluorescence intensities [140]. This method usually starts with preparation of reaction solution containing small molecular carbon precursors such as folic acid, vitamin C, and saccharose. Sometimes, additional surfactants are added to help to produce uniform disperse of precursors [141]. Such a method offers advantages including fast reaction rate, homogeneous heating of reactant solution, lower processing cost and higher yields [142]. Under microwave irradiation, the precursors are able to polymerase into particles. For example, in the preparation process of CQDs from L-ascorbic acid and β-alanine, four steps, namely polymerization, aromatization, nucleation and growth, were involved in the synthesis. Ascorbic acid and alanine first underwent intra-molecular dehydration at above 100 ℃ forming bigger sized polymeric particles. With increased temperature and time, aromatic clusters were then formed followed by nucleation burst, resulting in CQDs [143]. Furthermore, microwave treatment can be used in post-synthetic modification of CQDs. In a recent study, Huang et al*.* reported that microwave treatment can improve the optical properties of CQDs [144]. In this experiment, a batch of GQDs were first prepared through hydrothermal method. Subsequently, those GQDs were dispersed in distilled water and subjected to microwave treatment for 5 min in a conventional microwave oven. As a consequence, the GQDs exhibited stronger absorption peaks and the QY of GQDs increased from improved from 21.0% to 34.6% after treatment. This enhancement can be interpreted as the prolonged π-conjugated system and the suppression of non-radiative processes.

The hydrothermal method is another common method based on the “bottom-up” approach. It is generally considered as an environmentally friendly, controllable and cost effective method where chemical reactions take place between different carbonaceous precursors in hydrothermal reactor under high temperature and pressure [89, 145, 146]. Similar to the microwave-assisted irradiation method, the formation process of CQDs in this method usually is consist of four steps: decomposition, polymerization/aromatization, nucleation and growth [147]. Firstly, carbonaceous precursors such as glucose, fructose and amino acids undergo intermolecular dehydration and polymerization forming carbon skeleton and aromatic clusters. The nucleation of CQDs then takes place when the clusters reach its critical supersaturation point. Meanwhile, aromatic clusters diffuse towards particle surface resulting in the formation of functional groups on the surface of CQDs. Finally, as the reaction continues, the reaction intermediate convert into complete CQDs with a narrow particle size distribution. The properties of synthesized CQDs through hydrothermal method are dependent on reaction time, reaction temperature and reactant. For example, Qu et al*.* investigated the influence of different reaction conditions such as carbon sources, reaction time, and reaction temperature on QY [148]. They used citric acid and different organic amines as raw materials and synthesized a series of CQDs. In their study, the size of CQDs increased from 2.45 nm to 7.11 nm when the reaction time was extended from 2 to 24 h. The element analysis results showed that the amount of nitrogen atom doped into carbon core was increased with the reaction time and the QY was enhanced from 58 to 81% at the same time. Furthermore, they noticed that the resulting CQDs’ QY were sensitive to reaction temperature. The QY decreased at different degrees when the reaction temperature was higher or lower than the optimal condition. The choice of reactant could also influence the optical properties of CQDs. CQDs synthesized from citric acid and primary amine showed the highest nitrogen doping degree than those from secondary and tertiary amine and they exhibited the strongest photoluminescence intensity.

### Properties of carbon quantum dots

**Fig. 5:**
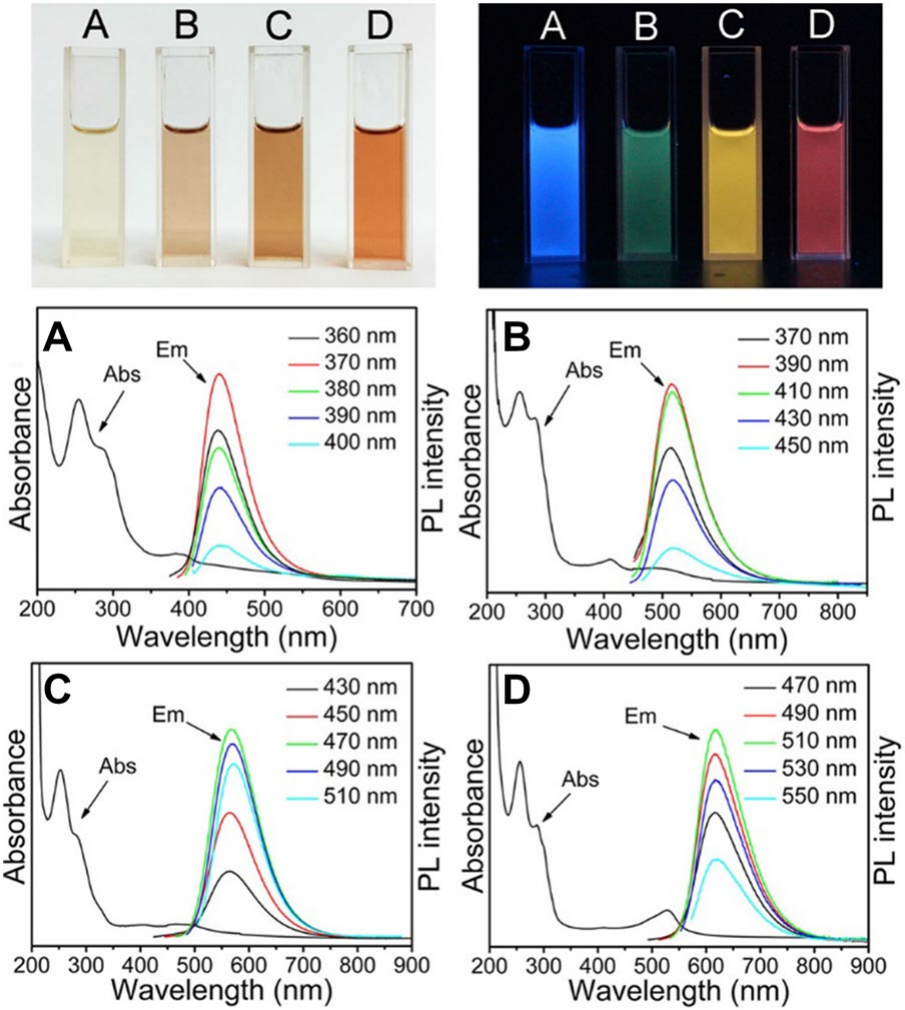
Top images are photographs of CQDs samples **A**–**D** in aqueous solution under daylight (left) and UV light (right). The bottom four graphs show their absorption curves and their PL emission spectra under excitation with light of different wavelengths [146]. Reproduced with permission. Copyright © 2016, American Chemical Society

As a promising type of functional nanomaterials, one of the most fascinating features of CQDs is their optical property. Since there are conjugation systems in the chemical structure of CQDs, a typical absorbance spectrum of CQDs exhibits optical absorbance mainly in the UV region with a tail extended to the visible range. For example, Ding et al*.* reported a one-pot syntheses method to fabricate full-color light-emitting CQDs. After purification via silica column chromatography, these CQDs exhibited excitation-independent luminescence from blue to red (Fig. 5). [146] Under UV excitation, CQDs can show fluorescent emissions. Generally speaking, the synthesized raw CQDs do not initially possess fluorescence properties. However, after surface passivation with polar moieties the CQDs can exhibit outstanding fluorescence properties [124]. In earlier researches, a excitation wavelengths dependent manner was presented for their emission intensity and wavelength [149]. The emission wavelength was moved toward longer wavelength region with the increase of excitation wavelength, and the emission could almost cover all visible region [150]. However, some recent researches have indicated that certain types of CQDs could emit light of specific wavelength which is independent of the excitation wavelength [146, 151]. As shown in Fig. 5, the emitting modes of some CQDs are free from the effect of excitation wavelength but differ with the variety of solvent.

The fluorescence properties of CQDs could also be impacted by some other factors. Generally, when the concentration of CQDs is increased, the dominated energy transition changes from π-π* energy transition to n-π* energy transition, resulting in a shift of emission wavelength to the longer wavelength region because of the enhancement of surface-surface interaction between CQDs [152]. Moreover, the surface energy level of CQDs would decrease leading to a lower fluorescence lifetime. In addition, the size of CQDs could influence the fluorescence properties of CQDs. In most cases, their photoluminescence wavelengths show a red-shift phenomenon when the size is increased [153]. At present, the luminescence mechanism of CQDs still remains unclear. Their luminescence phenomenon can be attributed to several reasons, including quantum size effect, crystal structure, surface state, surface passivation and functional group [154, 155].

In general, all synthesized CQDs need to undergo surface passivation or surface oxidation to obtain fluorophore modification. Thus, the surface of CQDs is usually medicated with oxygen containing functional groups and most CQDs are hydrophilic [156]. The hydrophilicity of CQDs also depends on reactant and synthetic method. The CQDs derived from soluble precursors tend to have high hydrophilicity since the functional groups of precursors are remained on the surface of CQDs during fabrication process. On the other hand, CQDs derived from insoluble precursors may also become water-soluble after certain surface passivation and modification. In a previous study, Pankaj et al*.* synthesized hydrophilic CQDs from hydrophobic candle soot [157]. In their study, the candle soot was treated with nitric acid resulting in a CQD product with carboxylic acid groups on the surface. Ethylene diamine was later added to convert the surface groups to amino groups.

Compared with traditional inorganic quantum dots, which contains heavy metal elements, CQDs generally exhibit better water solubility and biocompatibility, evidenced by plenty of studies and researches on their cytotoxicity [158, 159]. For example, Nair et al*.* synthesized GQDs through sonochemical method with intermittent microwave heating and then tested the cytotoxicity by using MTT assay with HeLa cell line [160]. In the high GQDs dose (1000 μg mL^−1^) treatment group, no apparent cytotoxicity (cell viability > 92%) was found. In another study, Yao and his co-workers evaluated the in vitro cytotoxicity of bare CQDs and ferritin loaded CQDs [161]. Both CQDs and ferritin loaded CQDs, at a high concentration at 200 mg mL^−1^, showed good biocompatibility to both normal breast cell lines (cell viability > 85%) and breast cancer cell lines (cell viability > 90%). The source of raw material that can be used to generate CQDs is extensive ranging from synthetic compounds to natural products. Some CQDs may also be already existed in the natural environment and interacted with human body. Wang et al. investigated the formation process and cytotoxicity of CQDs which are generated in baking lamb. During the baking process, proteins, lipids, and carbohydrates of lamb may undergo decomposition and carbonization processes and form CQDs at high temperature [162]. It was found that CQDs from high temperature baking lamb exhibited greater cytotoxicity that those from low temperature baking. However, in general, those CQDs have low cytotoxicity (with a viability more than 90% at a high concentration of 2 mg mL^−1^) and exhibited excellent biocompatibility under experimental conditions. On top of it, CQDs possess the potential of radical scavenging ability that can protect HepG2 cells from H_2_O_2_ induced oxidative damage [162].

Jia et al*.* evaluated the in vivo biocompatibility of CQDs by injecting CQDs to mice and recorded the change of body weights [163]. Their results demonstrated that after intravenous injection, the CQDs were mainly accumulated in tumor tissue, liver and kidney and were then gradually excreted from body within 14 days. The body weights were unchanged and there were no evident histological alterations demonstrating the excellent biocompatibility of CDs.

### Biomedical application of carbon quantum dots

Biomolecular detection has played an important role in disease diagnosis, clinical treatment and medical research, etc. In recent years, CQDs have been reported for the use in biomolecular detection. Qu et al*.* reported a new method to apply CQDs in detecting the concentration of dopamine in serum [164]. They produced CQDs with special catechol groups on surfaces that can react with Fe^3+^ by forming quinone groups, which quench CQDs. Upon addition of dopamine to the system, it could react with Fe^3+^ to prevent the reaction between Fe^3+^ and CQDs resulting in fluorescence recovery. In this way, dopamine concentration can be determined by drawing a standard curve of dopamine content and fluorescence changes. In this study, a detection limit of dopamine at 68 nM was achieved. An improved CQDs-based fluorescence method for dopamine detection was later reported by He et al. [165]. They developed a fluorescent probe by combining CQDs and gold nanoclusters, in which the CQDs acted as energy donor while the gold nanoclusters acted as energy acceptor. With the existence of dopamine, the fluorescence resonance energy-transfer was suppressed resulting in a fluorescent quenching of gold nanoclusters as well as a fluorescence recovery of CQDs. Thus, by measuring the intensities at two different emission wavelengths, the concentration of dopamine could be calculated. This detection method exhibited a low detection limit at 2.9 nM and a wide detection range between 5 and 180 nM. In addition to the small molecular detection, CQDs can also be applied in the detection of biomacromolecules and even bacteria. For instance, Yang et al*.* reported the CQDs-encapsulated breakable organosilica nanocapsules for the detection of pathogenic bacteria. This method is highly sensitive, and can detect S. aureus from 1 to 200 CFU mL^−1^ [166].

Due to their remarkable biocompatibility, water solubility and fluorescence behavior, CQDs have further been studied as novel bioimaging probes and have provided some fruitful results. Peng et al*.* used the hydrothermal method to synthesize functional CQDs that can bind to calcified bones with high affinity and specificity [167]. They conducted animal tests with zebrafish and found that the resulting CQDs successfully deposited in the abdominal cavity. Meanwhile the CQDs exhibited another potential to be applied in bone-targeted drug delivery. In a recent study, Liu et al. reported an improved bioimaging strategy that utilize red-emitting CQDs to achieve one-photon imaging and two-photon imaging [138]. These CQDs were synthesized from conjugated aromatic amine molecule under the action of oxidative radical reagents. Compared with short-wavelength near infrared (750–950 nm), long wavelength infrared photons allow deeper penetration into bio-tissues and thus enable deep and highly sensitive in vivo bioimaging. The resulting CQDs manifested featured red photoluminescence at 615 nm with high QY at 84% and a narrow emission linewidth. The results showed that CQDs can diffuse specifically in lysosome regions and bright luminescent regions were observed at the injected spot. In the two-photon bio-imaging mode, these CQDs exhibited up-conversion fluorescence which can emit featured red light upon the irritation of 1060 and 1100 nm infrared light. Hence, the imaging depth easily exceed 200 μm and the maximum penetration depth reached about 500 μm.

CQDs have also attracted increasing attentions in drug delivery and gene delivery. Feng et al*.* reported a CQDs-based drug delivery platform [168]. In their work, cisplatin was loaded to CQDs through electrostatic interaction and the complex could be released in tumor tissue within a low pH environment, possessing less side effects and high anti-cancer efficacy. Furthermore, elaborately designed CQDs can achieve targeting release and sustained release, thus increasing therapeutic index and reducing toxicity. Hua et al*.* designed a CQDs-based drug delivery platform for realizing nucleus-targeted drug delivery and photodynamic therapy [169]. The CQDs were fabricated using m-phenylenediamine and L-cysteine via the hydrothermal method. The resulting CQDs showed a universal nucleolus targeting ability to a variety of cell lines. The targeting ability can be attributed to the selectively binding with RNA molecule due to the specific surface chemistry of CQDs. By conjugating protoporphyrin IX with CQDs, the drug delivery platform was prepared. Compared with free protoporphyrin IX, the CQDs-based drug delivery platform demonstrated a comprehensive improvement in terms of tumor-homing performance, blood circulation, tumor retention, toxicity and anti-tumor efficiency. In another study, Singh et al. constructed CQDs-DNA hydrogels for sustained drug delivery and monitoring [170]. In the first step, the 5′-phosphorylated DNA molecule was used to functionalize CQDs forming CQDs-DNA complex. In the second step, by adjusting the solution pH, the conformation of DNA was changed leading to a sol to gel transition. Finally, doxorubicin (DOX) was encapsulated into CQDs-DNA through electrostatic adsorption. Due to the disruption of intermolecular i-motif structure of CQDs-DNA hydrogel in acidic pH, it can release drug in acidic cancer tissue. It was observed that the hydrogels could release 91% of the total encapsulated DOX at pH 6 through a span of 11 days while the release rate at pH 7.4 was much slower.

## Application of carbon quantum dots for virus detection

CQDs have been demonstrated as a superior nanomaterial possessing distinct electronic, optical, mechanical, chemical and thermal properties. In recent years, considerable efforts and developments have been made toward virus detection.

### Optical sensing

Optical biosensor is an analytical device which transduces a biological response into a measurable optical signal in terms of phase, amplitude, resonant momentum, absorption or quantity of light emitted [171]. Due to the unique characteristics of carbon-based dots, the optical biosensor, especially fluorescence biosensor based on CQDs and GQDs, have extracted extensive research interests in recent years. Compared with traditional dyes, CQDs display better optical properties in terms of broad and large Stokes shifts, narrow emission band, comparatively long lifetimes and higher molar absorption coefficients [172, 173]. Moreover, CQDs are facile to be modified via surface modifier absorption [125]. Owing to these above advantages, CQDs are suitable to be used as fluorescence detective probe. In the detecting process, the excitation and emission signal can be easily separated and thus the optical sensor which utilize CQDs probe is of low background noise and high sensitivity.

Liang et al*.* developed a ratiometric fluorescence biosensor composed of CQDs and cadmium telluride quantum dots (CdTe QDs) for serum human immunodeficiency virus (HIV) dsDNA detection [174]. In their work, 3-mercapropionic acid-coated CdTe QDs were first coupled with mitoxantrone (MTX), a synthetic anthraquinone drug that can intercalate with DNA, resulting in quenching of red fluorescence at 599 nm owing to the electron transfer between CdTe QDs and MTX. In such a case, only the fluorescence emission at 435 nm owing to green-emitting CQDs could be detected. However, in the presence of HIV dsDNA, the specific binding of MTX to dsDNA led to a dissociation between MTX and CdTe QDs. Owing to the electrical repulsion, CdTe QDs moved further away from MTX-ssDNA complex. Thus, the fluorescence emission at 599 nm was recovered. A fine linear relationship was observed between the dsDNA concentration in the range of 0–50 nM and I_599_/I_435_ (I_599_ and I_435_ represent the fluorescence emission intensity at 599 nm and 435 nm, respectively). The researchers further tested their biosensor with human serum samples. Both high recovery and low standard deviation were achieved.

**Fig. 6:**
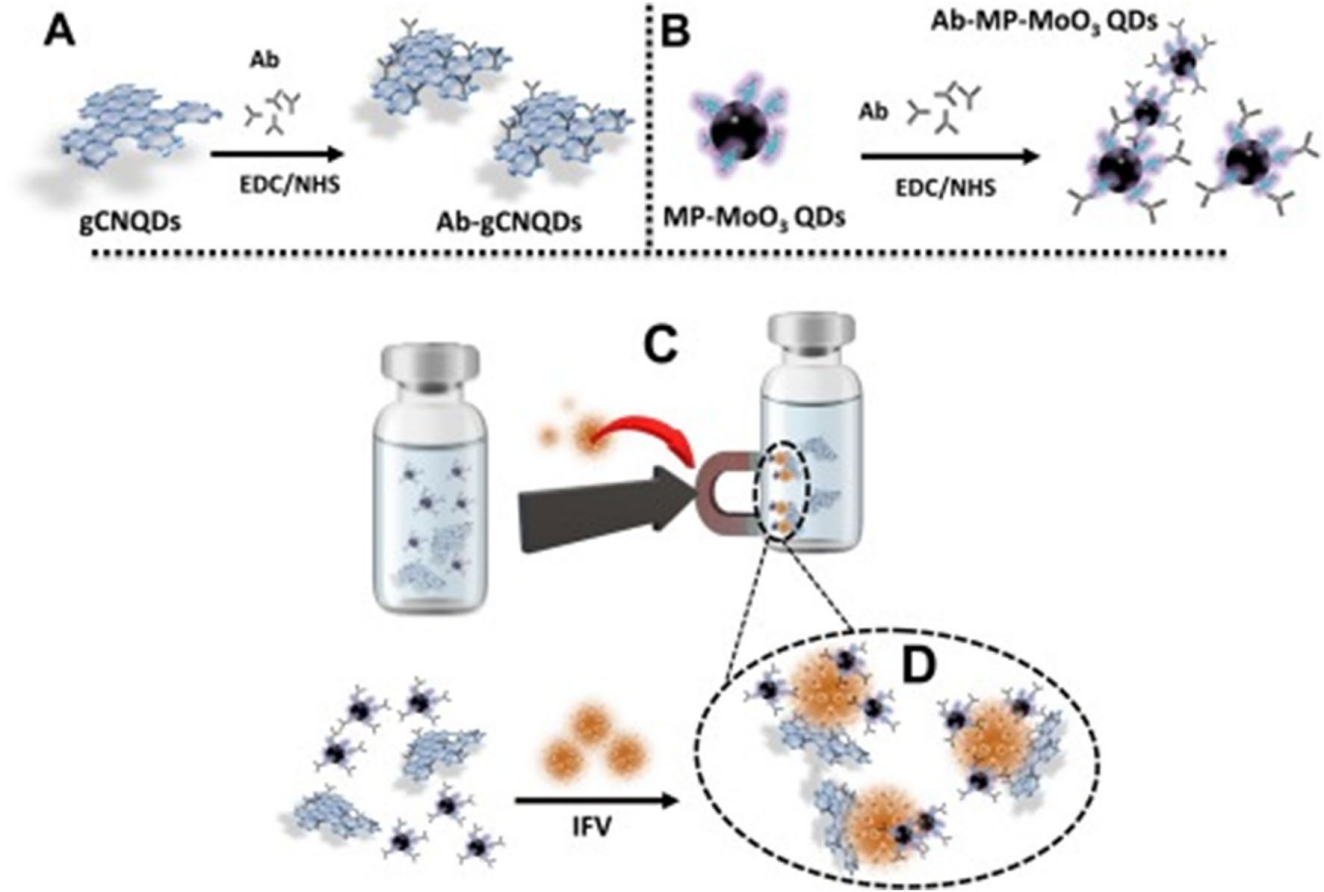
**A** Antibody conjugation to CQDs via covalent chemistry. **B** Antibody conjugation to MP-MoO_3_ QDs via covalent chemistry. **C** Magnetic separation and purification step upon target virus addition. **D** Core-satellite immunocomplex of CQDs and MP-MoO_3_ QDs in the presence of influenza virus [177]. Reproduced with permission. Copyright © 2020 Elsevier B.V

Researchers have found that the fluorescence signal of CQDs can be greatly enhanced by surface plasmon resonance (SPR) effect [175]. SPR effect is a resonant oscillation manifestation of conduction electrons on material surface induced by incident photons [176]. Based on this principle, a fluorescence detection platform composed of CQDs for virus detection was developed [177]. As shown in Fig. 6, magnetic-derivatized plasmonic molybdenum trioxide quantum dots (MP-MoO_3_ QDs) and fluorescent CQDs were first synthesized. The MP-MoO_3_ QDs have a single-layered morphology with few-crystalline-structure and oxygen vacancies. Herein, MP-MoO_3_ QDs possess tunable localized SPR effect. The CQDs were prepared from graphitic nanosheets through hydrothermal treatment. The absorption spectra of synthesized MP-MoO_3_ QDs overlapped the fluorescence spectra of CQDs. When these two QDs closed up, due to the energy transfer process and SPR effect, the fluorescence of CQDs was significantly enhanced. In the detection process, MP-MoO_3_ QDs and CQDs were first immobilized with virus targeting antibody, respectively. After incubation with influenza A virus, the CQDs bound to virus and formed a core-satellite network. Since MP-MoO_3_ QDs were close to CQDs, an increased fluorescence signal was observed. By detecting the change of fluorescence intensity, the content of virus can be quantified. The detection limit of this method could reach 45 PFU mL^−1^ and a wide detection range up to 25,000 PFU mL^−1^.

In a similar study, Achadu et al*.* reported a fluoro-immunoassay for influenza virus detection. They first synthesized sulfur-doped graphitic carbon nitride QDs (S-gCNQDs) via solvothermal treatment [178]. After that, anti-human influenza virus A antibodies were attached to the surface of S-gCNQDs and Ag_2_S nanocrystals. Influenza A virus isolated from biological samples could be captured on S-gCNQDs and Ag_2_S nanocrystals forming a nano-sandwich complex. In view of metal-enhanced fluorescence effects that occur when semiconductor nanostructures close to fluorophores, the optical property of fluorophores alters. In this assay, the local optical field induced by electronic interaction between Ag_2_S nanocrystals and S-gCNQDs made a contribution to this phenomenon. Thus, the formation of nano-sandwich complex resulted in a dramatic increase of fluorescence intensity and the fluorescence variation was proportional to virus content. By establishing a standard curve between influenza A virus and fluorescence intensity, the virus from unknown sample could be detected and quantified. This method has merits of high sensitivity and high-speed inspection. It allows to detect influenza A virus from human serum in the range from 10 fg mL^−1^ to 1.0 ng mL^−1^ with a detection limit of 5.5 fg mL^−1^ and the whole testing process can be completed in 15 min. This detection method not only provides a highly sensitive detection method for virus but also a new direction for designing QDs based nanostructure with strong luminescence emission.

### Electrochemical sensing

Electrochemical biosensor is one of the earliest biosensor devices and can be traced back to early years of the twentieth century [179, 180]. A typical electrochemical biosensor usually consists of a recognition mode, a transduction mode, and a signal processing mode, translating the biochemical signal into a recognizable electrical signal [181]. In such a biosensor, the electrodes are usually functionalized with biomacromolecule, such as enzyme, antibody and DNA. Hence, a biochemical signal can be generated by the molecular recognition event or enzyme catalyzed reaction [182]. However, a number of studies have indicated that enzyme based electrochemical biosensor faces problems of insufficient stability, lower reproducibility and influence of oxygen limitation [183].

**Fig. 7:**
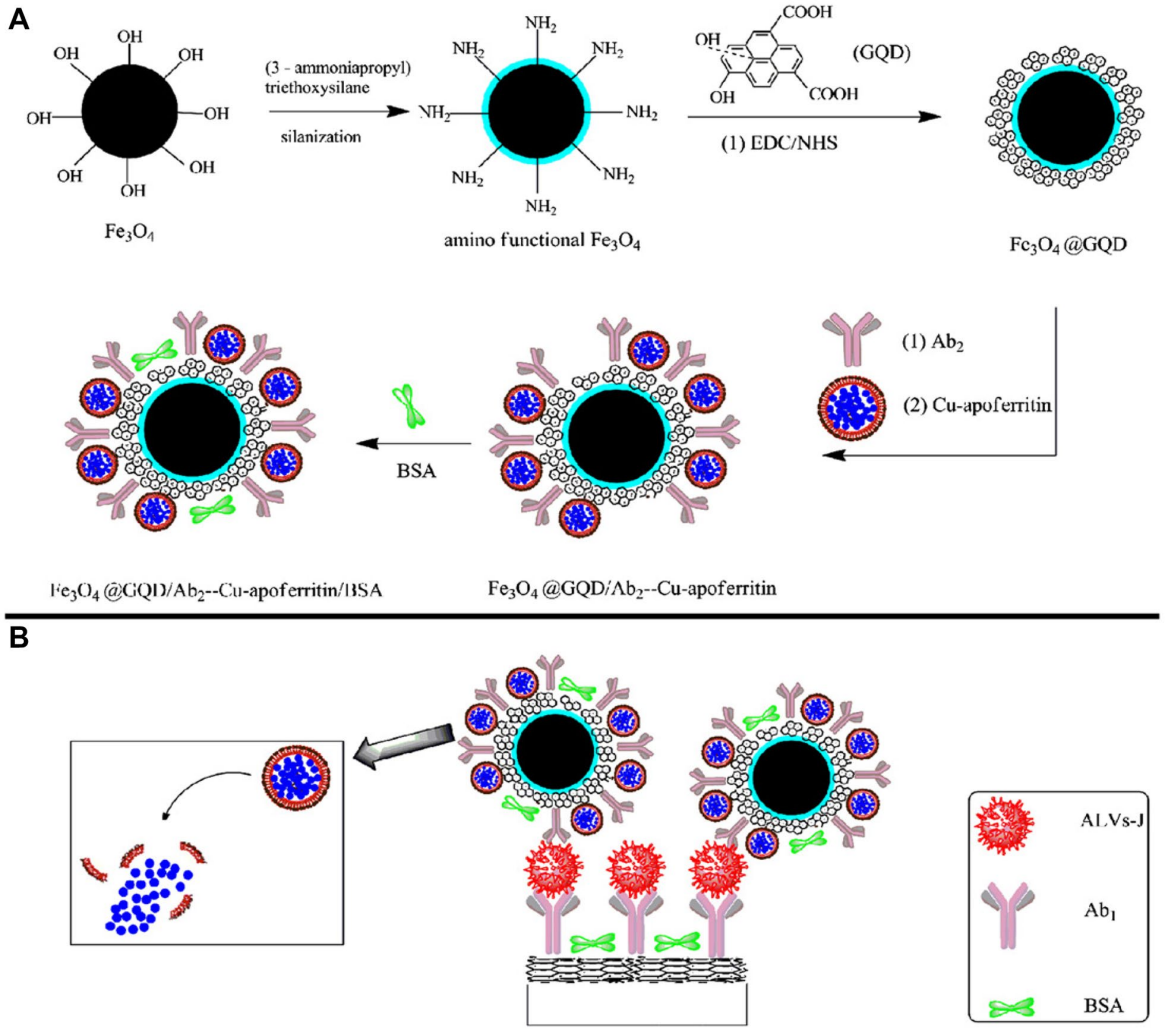
Schematic representation of the construction of detection probe (**A**) and detection principle of GQDs-based nano system (**B**) [184]. Reproduced with permission. Copyright © 2013 Elsevier B.V

Non-enzymatic electrochemical biosensors are considered to be a prospective new field which is possible to overcome these limitations. CQDs have a large surface area which could allow various detection events to occur simultaneously. Furthermore, some CQDs-based electrochemical biosensor have exhibited excellent performance in terms of high electro catalytic activity, extensive operating potential range and high electrical conductivity. Wang et al*.* successfully developed a sandwich-type electrochemical immunosensor for detecting avian leukosis virus subgroup J [184]. In their work, the Cu-apoferritin nanoparticles were synthesized as electroactive probes and GQDs were synthesized through chemical oxidation method. The surface of GQDs was functionalized with abundant of carboxylic groups providing a large number of binding sites for capturing antibodies as well as electroactive probes. As can been seen in Fig. 7, the amino-modified magnetic iron oxide nanoparticles were first prepared acting as the matrix for loading GQDs. Then the GQDs were immobilized on the surface of magnetic iron nanoparticles, followed by coupling anti-avian leukosis virus subgroup J antibodies and Cu-apoferritin nanoparticles. During the fabrication of electrochemical immunosensor, the glassy carbon electrode (as the working electrode), was polished and coated with GQDs layer. After that, anti-avian leukosis virus subgroup J antibodies were immobilized on the electrode. In the case of existence of virus, virus targeting antibodies captured the virus forming a sandwich structure. Under acidic conditions, the protein cage of Cu-apoferritin dissociated into subunits releasing Cu, and the Cu atom was further oxidized into Cu^2+^ generating a measurable redox potential peak. Due to the electroactive probes are specifically bonded to virus, the amount of released Cu atom is directly proportional to the virus quantities. Thus, the concentration of virus can be evaluated via redox peak currents with a detection limit down to 115 TCID_50_ mL^−1^ and a dynamic detection range between 10^2.08^ and 10^4.50^ TCID_50_ mL^−1^.

Recently, an improved CQDs-based electrochemical sensor, namely pulse-triggered ultrasensitive electrochemical sensor, was reported by Chowdhury et al*.* for hepatitis E virus (HEV) detection [185]. They elaborately designed the electrode and functionalized it with different layers of materials. In the first step, the researchers utilized aniline monomer as precursor to coat the bare glassy carbon electrode with a conducting polyaniline layer which exhibits excellent long-term stability and provides interaction sites between matrix and nanoparticles [184]. In the next step, nitrogen and sulfur co-doped GQDs and gold embedded polyaniline nanowires were synthesized, separately. The doping of N atom to GQDs offers enhanced electrochemical properties and the sulfur atom-doping in GQDs provides binding sites to gold nanoparticles. The synthesized GQDs were further modified with anti-HEV antibodies to endow them with virus targeting ability, and then they were conjugated to gold embedded polyaniline nanowires through Au–S bonds. Finally, the CQDs@gold embedded polyaniline complex was drop-cast on the polyaniline coated electrode. The composite coating on electrode could optimize the electrochemical response and provide specific binding area for capturing HEV. By conducting electrochemical impedance spectroscopy analysis, it was found that after the loading of HEV, the value of charge transfer resistance was increased dramatically. This phenomenon was attributed to the introduction of nonconducting virus to the conducting surface of GQDs and gold embedded polyaniline nanowires. The calibration curve was obtained by plotting the data of HEV concentration and change of charge transfer resistance between the virus-loaded electrode and the control group. In a proof-of-concept study, serum samples were tested. Despite some interferences raised by the disruptor in serum, the sensor exhibited a good linear relationship and showed an impressive capability of detecting trace virus. A wide detection range between 10 fg mL^−1^ and 100 pg mL^−1^ of serum HEV was reported. Compared with some other virus detection methods, the detection limit in this biosensor is tenfold lower [185].

### Other bioassay methods

CQDs have also been applied as functional nanomaterials displacing or enhancing the function of traditional detective probes in bioassays for virus detection. For example, Ahmed and his co-workers reported an optoelectronic sensor for fowl adenoviruses detection [186]. In their study, gold nanobundle film and GQDs were combined together to generate electric signal under ultraviolet–visible light irradiation. The nanohybrids of gold film and GQDs provided superior optical performance for detecting virus.

Kurdekar et al*.* reported a paper-based immunoassay platform which applied CQDs to detect HIV antigen [187]. The platform took a detection mode similar to sandwich ELISA. The primary antibody was immobilized on the test zone of paper microplate. The CQDs were synthesized and functionalized with streptavidin. Upon binding of HIV antigen to primary antibody, biotinylated detector antibody was added to form a sandwich structure. Finally, CQDs were introduced and bound to the sandwich complex through biotin-streptavidin conjugation. The quantification analysis was achieved based on fluorescence intensity. Compared with the conventional ELISA test, a sensitive detection ranging from 250 pg mL^−1^ to 10 μg mL^−1^ was reported in this work.

## Application of carbon quantum dots for inhibition and treatment of viral infection

In recent years, along with more development and innovation, nanotechnology has revolutionized our lives, greatly redefining many biomedical/pharmaceutical fields such as diagnosis, therapeutics, drug delivery, and formulation [11]. Due to their distinct and advanced properties, the feasibility of CQDs in drug deliver, gene delivery, phototherapy and radiotherapy have been widely studied and verified. Some studies have revealed that certain CQDs have low toxicity to mammalian cells and high antiviral activity [188].

### Interfering virus entry and uncoating

The life cycle of viruses with animal hosts can be generally divided into three steps: (1) viral entry and uncoating; (2) viral replication and assembly; and (3) viral release [24, 189]. The first step is generally considered to begin upon the attachment of invading virus to the host cells [190]. After that, virus can inject their genetic material into host cell or the virus particle can penetrate a host cell membrane through a endocytic pathway [191]. Since viral replication can only take place when virus parasitize living cells, stopping virus from binding and invading to cells is regarded as one effective strategy to prevent virus infection. Some CQDs have been demonstrated with an ability of virus prevention/inhibition. Their inhibition efficiency against virus depends on the surface chemical structure, composition, size and shape of CQDs [188]. For example, it has been reported that boronic acid modified CQDs were able to block the interaction between cells and virus thus suppressing virus invasion [22]. Fahmi et al. reported that boronic acid modified CQDs could act as an entry inhibitor to prevent HIV infection [192]. According to a previous study, boronic acid could selectively act on glycopeptides and glycoprotein leading to a specific interaction with HIV [192]. Their CQDs were produced first by pyrolysis of citric acid. Since they possess an abundance of surface groups such as hydroxyl and carboxylate groups, carboxyl phenylboronic acid could be grafted on the surface. The resulting CQDs were then tested against HIV in MOLT-4 cells. They could bind to gp120, a kind of glycoprotein expressed on HIV envelop responsible for the attachment to human target cells, hindering the interaction between HIV and MOLT-4 cells membrane. Under high concentration of CQDs, the boronic acid sites of CQDs reacted with 1,2-cis diols sites on gp120, forming tetravalent boronate diester cyclic complex. Thus, the boronic acid modified CQDs exhibited excellent antiviral ability with an IC_50_ value at 26.7 mg mL^−1^.

More interestingly, a benzoxazine monomer-derived CQD was recently reported as a broad-spectrum agent to block viral infection against some life-threatening flaviviruses (Japanese encephalitis, Zika, and dengue viruses) and non-enveloped viruses (porcine parvovirus and adenovirus-associated virus [23]. The broad-spectrum antiviral ability is attributed to the binding of CQDs to virus which interfere the interaction between virus and cells.

In addition to targeting and bind virus, CQDs can also be bound to cell membrane surface to block the virus-cell interaction. In a previous study carried out by Barras et al*.*, boronic acid modified CQDs from hydrothermal method were demonstrated to against herpes simplex virus type 1 (HSV-1) infection [22]. Compared with other reported antiviral nanoparticle-based inhibitors (e.g. tannic acid modified Ag nanoparticles, dextran sulfate and poly-l-lysine), the synthesized CQDs exhibited higher antiviral effects. To further reveal the antiviral mechanism, the researchers evaluated the zeta potential changes of cells and virus before and after incubating with CQDs. The co-culture of negative charged CQDs did not reduce the zeta potential of virus while the incubation of CQDs to mammalian cells resulted in a significant change in zeta potential. Very interestingly, the authors further studied the role of boronic acid groups in viral prevention. Fructose was applied to convert boronic acid groups to a five-membered cyclic ester, followed by a viral inhibition test. CQDs after fructose treatment were found remaining high antiviral ability. It thus suggested that the interference of virus invasion could still be implemented by binding CQDs to cells’ surface receptors, without a need of boronic acid groups.

**Fig. 8:**
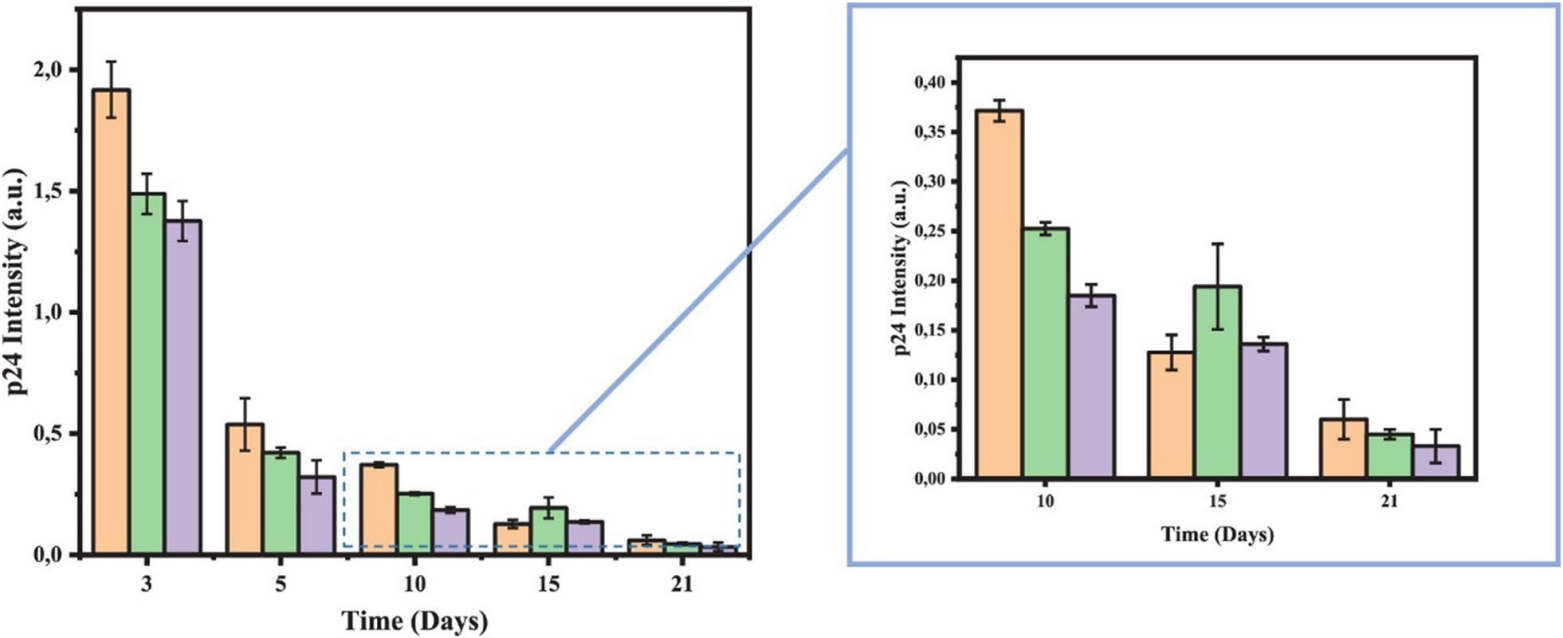
p24 Intensity of HIV-1 viral post-infection to MOLT-4 cells in the presence of pure CQDs (orange bars), boronic acid modified CDs (green bars), and boronic acid modified CQDs + Duviral (21 mg, purple bars). All of the data is presented as mean ± SD [193]. Reproduced with permission. Copyright © 2020, American Chemical Society

CQDs are further able to be combined with some existing antiviral drugs for improved therapeutic effects. Aung et al*.* adopted the hydrothermal method to synthesize CQDs having boron acid sites and graphene-like structures [193]. In consist with some previous studies, again the boron acid modified CQDs showed superior performance in inhibiting HIV invasion. This strong inhibition ability was attributed to the binding of boron acid sites to gp120 through H-bonding and covalent bonding [193, 194]. In order to achieve a better therapeutic effect, the resulting CQDs were further combined with durival, a multicomponent drug used to interfere the function of nucleoside reverse transcriptase, to treat test samples. As can been seen in Fig. 8, the combined complex exhibited a better antiviral result. As it is well known, HIV is a kind of retrovirus that targets T cells as host and is easy to evolve drug resistance. Hence by using effective remedy at an early stage can efficiently improve survival rate and life expectancy [195]. The combination of cocktail of various drugs that act on different viral targets is known as a highly active anti-retroviral therapy (HAART) which is one of most effective methods to combat HIV [196, 197]. HAART can significantly reduce virus count in the bodies, avoid drug resistance, reduce the risk of complications and recover immune function [198]. In light of this concept, CQDs exhibit a great potential for HAART.

### Interfering virus biosynthesis

Upon viral genome entry to host cells, the transcription or translation of viral genome is commenced followed by biosynthesis of viral proteins and genome. In this process, a large number of viral particles are produced and assembled. In addition to interfere virus attachment and penetration, CQDs are able to combat viral infection by inhibiting virus biogenesis. Iannazzo et al*.* investigated the feasibility of using drug-loaded GQDs as non-nucleoside reverse transcriptase inhibitors (NNRTI) to treat HIV [199]. They synthesized mono-dispersed GQDs by using multi-wall carbon nanotube (MWCNT) as carbon source through prolonged acidic oxidation and exfoliation. The obtained GQDs were modified with carboxylic groups on the surface and the dispersion stability was further enhanced. After that, two NNRTI, namely CHI499 and CDF119, were anchored on the surface of GQDs through esterification, respectively. It was found that compared with free drugs, GQD conjugated with NNRTI showed significant combination therapeutic effect. The conjugation of CQDs with CHI499 greatly enhanced the antiviral efficacy (IC_50_ of 0.67 versus IC_50_ of 0.09 ± 0.12, with and without GQDs) while the conjugated complex of CDF119 and GQDs showed a reverse trend (IC_50_ of 4.05 ± 0.33 versus IC_50_ of 43.3 ± 17, with and without GQDs). This result could be attributed to the chemistry structure of GQDs and NNRTI. In comparison to the imide bond presented in the GQDs-CDF119 complex, the amide bond presented in the GQD-CHI499 complex is easier to be broken down due to the displacement of the sulfonamido leaving group, thus leading to an easier drug release in infected cells. Moreover, in addition to acting as a drug loading platform, CQDs also exhibited a certain level of antiviral activity. The polycarboxyl structure of GQDs could inhibit the activity of reverse transcriptase by blocking the virus binding to a cell. In cell culture media, drug-loaded GQDs showed better dispersibility and stability because the existence of organic moiety prevents the interaction between GQDs and salts, ions and biomolecules.

In another study, Ju et al*.* designed a CQDs gene delivery platform for treating Kaposi’s sarcoma-associated herpesvirus (KSHV) infection [200]. The resulting CQDs were functionalized with antisense locked nucleic acid (LNA) oligonucleotides. When CQDs were absorbed by cells, the LNA oligonucleotides bound to specific virus RNA leading to a degradation of virus RNA in a RNase H-mediated manner. In this way, the transcription of virus genome was prevented.

Recently it has been found that some antiviral compounds, as the precursor, can be used to synthesis antiviral CQDs. Tong et al*.* reported a method to synthesis glycyrrhizic-acid-based CQDs (Gly-CQDs) with high antiviral ability [20]. They used glycyrrhizic acid, a traditional Chinese herbal medicine possessing antiviral immunoregulation activity, as the precursor to synthesize CQDs via hydrothermal method [200, 201]. Gly-CQDs exhibited high antiviral effects that inhibited the proliferation of virus by approximately 5 orders of magnitude. In this study, the antiviral activity of Gly-CQDs could be activated through the following mechanisms: (1) inhibiting virus invasion and replication; (2) inhibiting virus proliferation; (3) inhibiting virus-induced reactive oxygen species (ROS) production; (4) regulating the expression of antiviral genes; and (5) stimulating interferon production. Based on the transmission electron microscope (TEM) and Fourier-transform infrared spectroscopy (FTIR) results, it was found that Gly-CQDs inherited most of the functional groups from glycyrrhizic acid and had a larger surface area which facilitated a stronger binding with the virus. In addition, the poor water solubility and serious side effects, which are normally associated with glycyrrhizic acid, were diminished when Gly-CQDs were used.

**Fig. 9:**
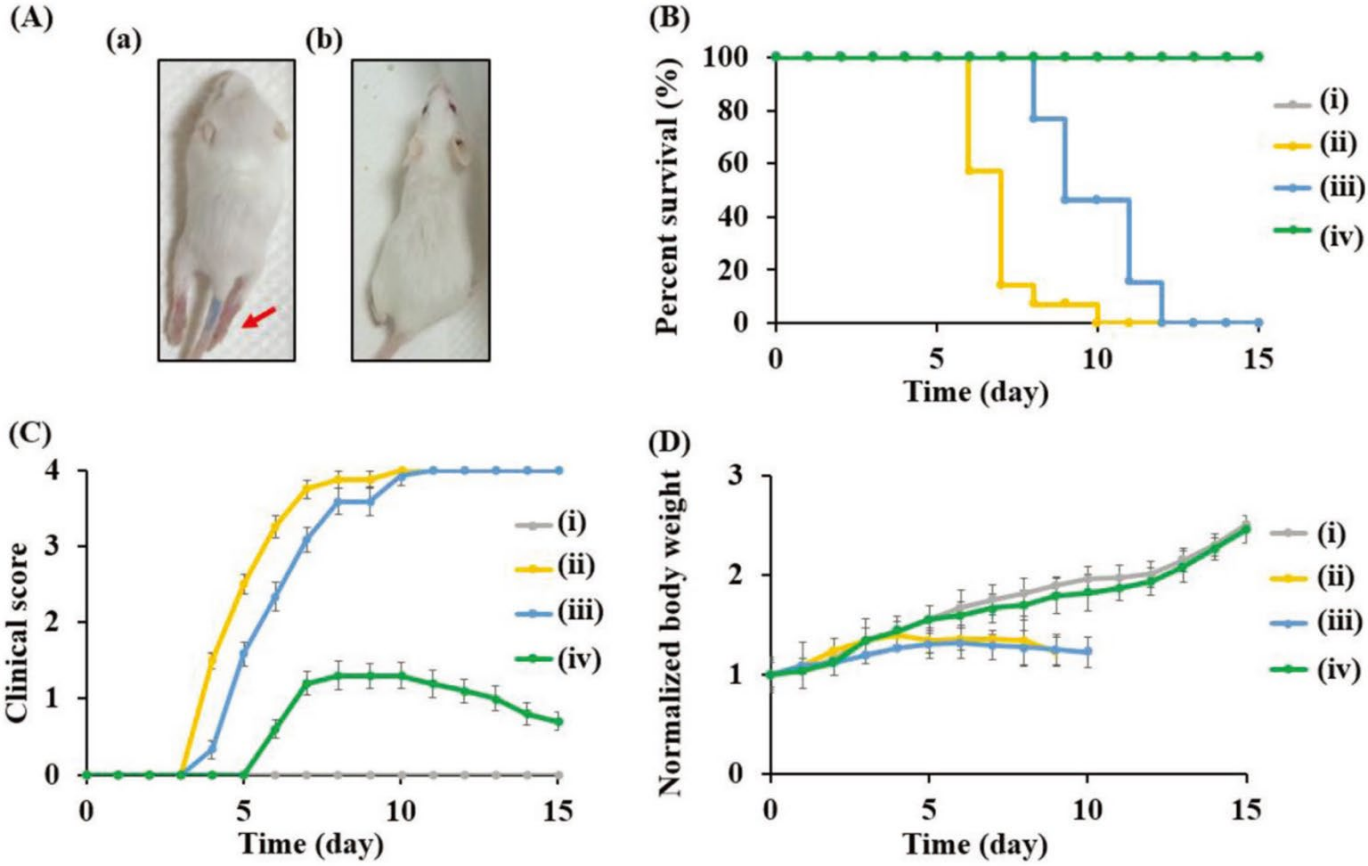
**A** Representative photographs of mice on day 7 post-infection (a) without and (b) treated with Cur-CQDs-180 (25 mg kg^−1^). The red arrow indicates apparent limb paralysis in the untreated infected mice. **B** survival rates, **C** clinical scores, and **D** body weights of (i) newborn mice without infection and (ii−iv) newborn mice intraperitoneally injected with (ii) PBS, (iii) curcumin (25 mg kg^−1^), or (iv) Cur-CQDs-180 (25 mg kg^−1^) followed by intraperitoneal injection of EV71 at the dose of 2 × 10^6^ PFU. A group of uninfected mice treated with sterile PBS was used as an uninfected control. The curcumin or Cur-CQDs-180 treatment was performed every 12 h daily for seven consecutive days. The mice were monitored for 15 d [202]. Reproduced with permission. Copyright © 2019 WILEY‐VCH Verlag GmbH & Co. KGaA

In another study, Lin et al*.* reported a kind of antiviral CQDs to against enterovirus by using curcumin as the precursor [202]. Curcumin is a natural compound and has demonstrated with an antiviral ability [203]. CQDs were synthesized by using a simple one-step dry heating method. In their study, curcumin was heated in a muffle furnace at 120–210 °C for 2 h to yield orange or brown residues followed by ultrasonication. After that, purified curcumin-based CQDs (Cur-CQDs) were obtained through centrifugation and dialysis. At high temperature, curcumin undergoes dehydration and condensation, followed by pyrolysis and carbonization, resulting in the sp^2^ hybridized carbon core. A small portion of curcumin or polymer-like curcumin was preserved on the surface of Cur-CQDs. Compared with curcumin, Cur-CQDs exhibited lower cytotoxicity and enhanced antiviral activity. As shown in Fig. 9, all virus infected mice without Cur-CQDs treatment were dead within 12 days. By contrast, over 95% of infected mice with Cur-CQDs treatment survived for at least one month. The immunological analysis suggested that Cur-CQDs could inhibit virus infection by interfering both virus attachment and replication. The superior antiviral activity of Cur-CQDs to curcumin can be interpreted as the change of chemical structure during the synthesis process. According to the mass spectrometry analysis, there were more active groups such as guaiacol, anisole and 1-hexatrienium on the surface of Cur-CQDs. Cur-CQDs’ good hydrophilicity and high density of antivirally active moieties may further help to improve their antiviral activity.

### Boosting immune response to virus

**Fig. 10:**
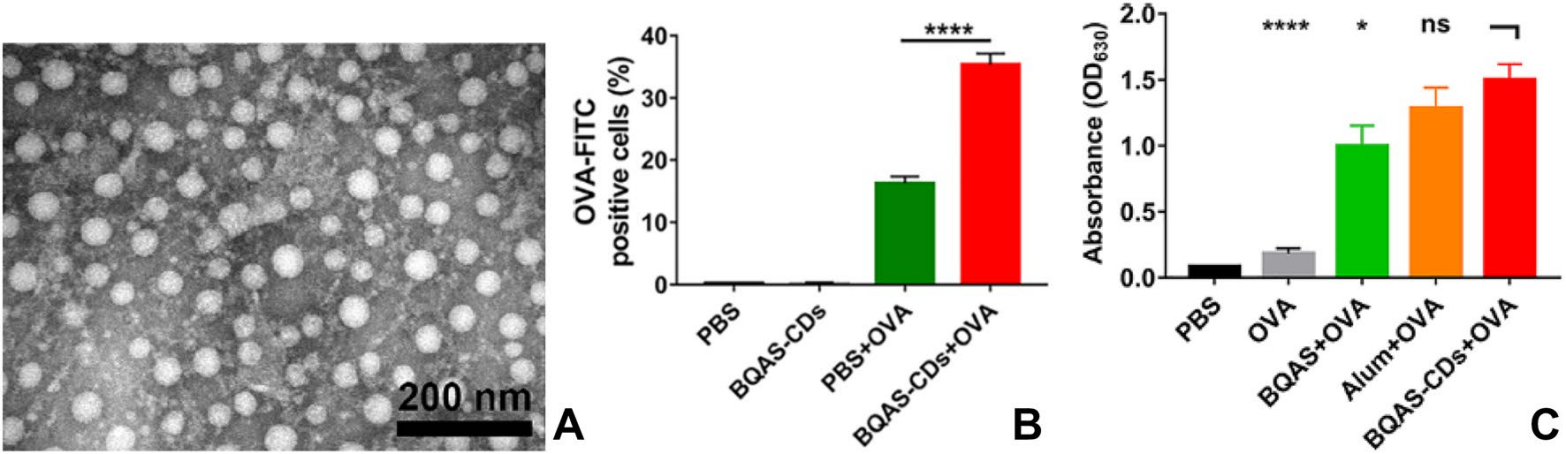
**A** TEM image of OVA-CQDs complex. **B** Percentage of positive BMDC cells after incubating with OVA and OVA conjugated CDs. **C** ELISA result of IgG concentration in sera of BALB/c mice intramuscularly immunized with different adjuvants: BQAS, alum, or CQDs [204]. Reproduced with permission. Copyright © 2020, American Chemical Society

The immune system is critical to the body's rebellion mechanism against viral infection. In recent years, many studies have revealed that CQDs can act in the immune system and enhance specific immunity. In a previous study, Huang et al*.* reported that quaternate cationic CQDs could act as an adjuvant to promote antigen presentation and induce robust immune response [204]. In this study, the positively charged CQDs were synthesized from hydrothermal method using bi-quaternary ammonium salt (BQAS) as raw material. Ovalbumin (OVA), as a model antigen, was then absorbed to CQDs through physical adsorption (Fig. 10A). In order to trigger a sustained immune response, antigens normally should be processed by antigen-presenting cells (APCs) and then APCs can activate T cells inducing specific immune response. However, small soluble antigens always face the problem of weak immunogenicity and are hard to be ingested by APCs. In comparison, it was observed that OVA bonded CQDs could yield stronger cellular uptake (Fig. 10B). Moreover, a stronger immune response was triggered by the resulting CQDs in this study. Mice which were immunized with OVA-CQDs complex secreted more OVA-specific IgG, about 60-fold higher than those treated with OVA only (Fig. 10C). The antibody secretion could last for more than 8 weeks. In addition, CQDs adjuvant could activate stronger cell mediated immunity. According to the results of flow cytometric analysis, it was found that OVA-CQDs treated mice exhibited more OVA-specific CD4^+^ and CD8^+^ T cells proliferation, and meanwhile a 1–2 fold increased proliferation of splenocytes was observed. Hence, CQDs adjuvant can stimulate both humoral immune and cellular immune. However, the mechanism of these effects still remains undefined.

In another study, some modified CQDs were reported showing an immunoregulatory capacity [205]. CQDs were first obtained from hydrothermal method starting from glucose and tetraethylenepentamine. In the next step, ricin toxin binding subunit B (RTB), one of the subunits of the ricin protein, was loaded to CQDs by physical absorption. The CQDs-RTB nanoparticles were then added to macrophages. As a result, the NO production of macrophages was increased in a dose-dependent manner. Furthermore, these cells could secrete more cytokines, TNF-α and IL-6. Compared with free RTB, CQDs-RTB nanoparticles showed a superior performance in modulating immune activity. This may be attributed to the larger size of CQDs-RTB nanoparticles since particles which have a comparable size to pathogens are more readily to be recognized and internalized by APCs.

CQDs are also able to exert anti-vital effects by directly boosting immune reaction to virus infection. Du et al*.* synthesized some CQDs using PEG-diamine and ascorbic acid as the carbon source via hydrothermal method [206]. Pseudorabies virus (PRV) and porcine reproductive and respiratory syndrome virus (PRRSV) were chosen as models of DNA virus and RNA virus respectively. The synthesized CQDs showed antiviral activity to both PRV and PRRSV by activating type I interferon responses. As a kind of glycoprotein with powerful antiviral activity, type I interferon (IFN-a and IFN-b) initiates intracellular signaling pathway leading to enhanced expression of IFN-stimulated genes [207, 208]. Upon injection of CQDs, an increased interferon-related mRNA expression was observed, indicating that the successful inhibition of virus replication was resulted by activating type I interferon responses.

## Carbon quantum dots and COVID-19

Since early of 2020, SARS-CoV-2 (severe acute respiratory syndrome coronavirus 2) has infected more than 170 million people and almost every country in the world [209]. The numbers of infection and death are still increasing quickly to date. As a response, more and more techniques and products associated with nanotechnology and nanomedicine have been extensively exploited and developed to against COVID-19 (coronavirus disease 2019) in the following areas: rapid point-of-care diagnostics, surveillance and monitoring, therapeutics, and vaccine development [210].

**Fig. 11:**
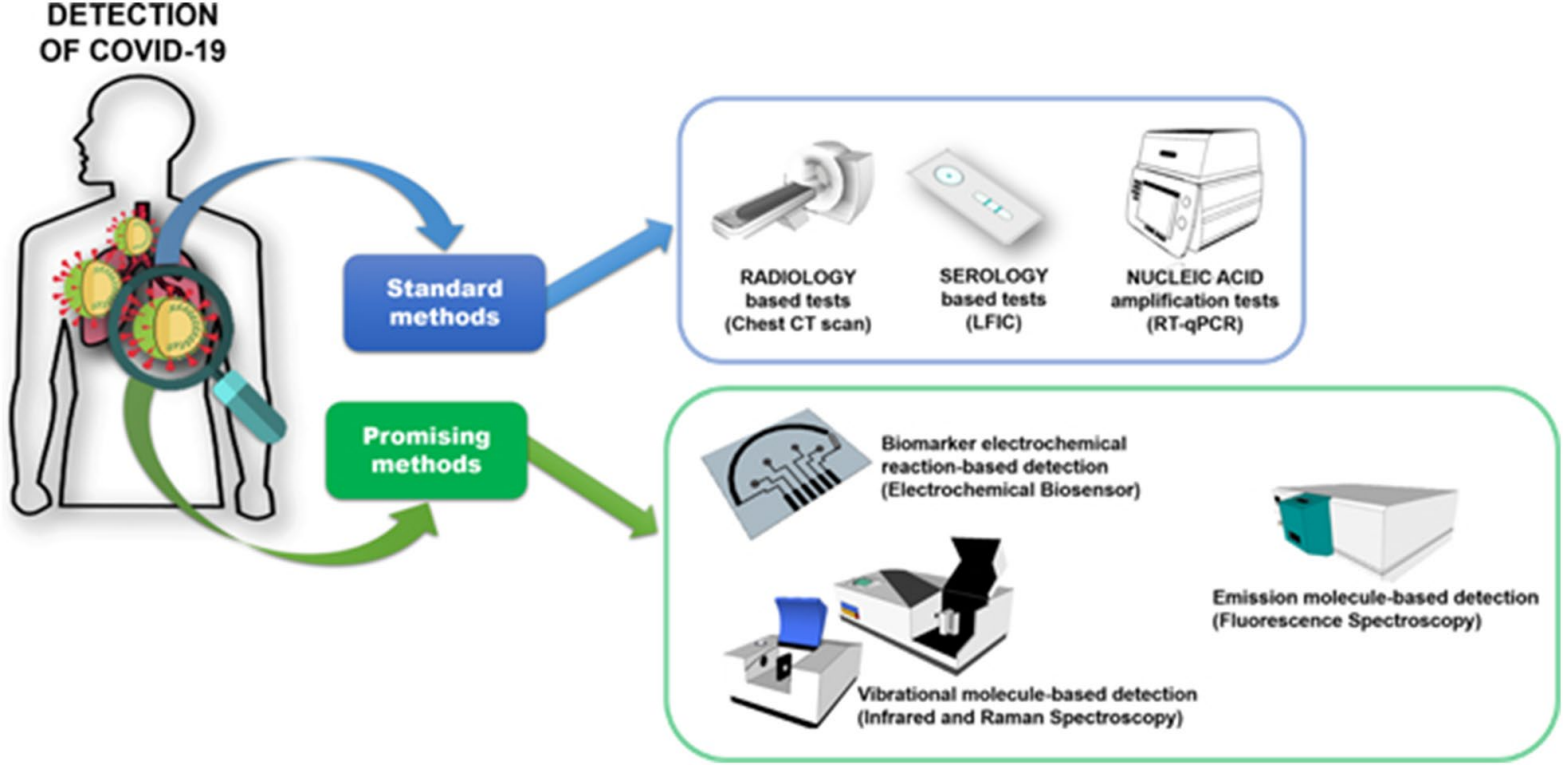
Schematic illustration of various detection methods for SARS-CoV-2 [219]. Reproduced with permission. Copyright © 2021 IOP Publishing Limited

Misdiagnosis and delayed diagnosis are common problems in healthcare. Thus early, rapid and accurate diagnosis is crucial to enhance survival rate by helping to identify patients earlier and offer treatments in time. However, the infection of SARS-CoV-2 sometimes shows no symptom or only some nonspecific and minor symptoms such as cough, fatigue, sputum production and shortness of breath [211]. Hence, there is a challenge for developing sensitive, early, rapid and specific COVID-19 diagnostic methods. The current commonly applied diagnostic method are CT (computerized tomography) scans, serological techniques and nucleic acid amplification test amplification test (NAAT) [212, 213] (Fig. 11). Recently, it has been reported that compared with syndromic testing and CT scans, molecular techniques are more suitable for accurate diagnosis [214]. The current molecular assay for SARS-CoV-2 mainly involves nucleic acid and serological based detection. The former approach usually utilizes nucleic acid testing techniques represented by reverse transcriptase real-time polymerase chain reaction (RT-qPCR) to detect RNA molecule of SARS-CoV-2. The latter approach uses immunological methods represented by enzyme-linked immunosorbent assay (ELISA) to detect antibody and antigen in serum. The nucleic acid testing techniques are regarded as more sensitive and effective for COVID-19 detection and are generally preferred to be used in detecting early viral infections since SARS-CoV-2 is an RNA virus and the reverse transcription of viral genome can happen in the early stage of infection [215]. However, nucleic acid testing of COVID-19 also faces some knotty problems. The NAAT is highly dependent on lab and related personnel. Any misplay during sample processing may lead to false negative results. It has also been reported that detection rate of NAAT is affected by the source of samples. For example, to the same group of patients, the rate of PCR detection of bronchoalveolar lavage fluid sample is much higher than that of stool samples (93% versus 29%) [216]. Besides, due to reduction of virus reproduction in the late stage of disease, virus RNA is always difficult to be detected and thus may arise false detection results. As a comparison, the serological based detection has high specificity and is easy to operate. In resource-limited labs, the serological based detection is easier than NAAT for large-scale detection [217]. Furthermore, the serological based detection can provide more information on both active and past infections and help the analysis of infection in the population. However, the serological based detection is impractical for early detection [218].

CQDs-based biomacromolecule detection have shown some excellent performances excellent feasibility in point of care testing for virus [219]. Recently, Qaddare et al*.* elaborately designed a CQDs-based fluorescence resonance energy transfer platform to achieve trace detection of virus DNA [220]. CQDs were synthesized from histidine through hydrothermal method. The obtained fluorescent CQDs were conjugated with DNA (CQD-DNA) and then bound to gold nanoparticles/graphene oxide composite which act as the fluorescence quencher. When they got in contact with HIV DNA, the conjugated DNA on the surface of CQDs was hybridized to HIV DNA and the binding interaction between CQD-DNA and gold nanoparticles/graphene oxide composite was disrupted and inhibited, resulting in a fluorescence recovery of CQDs. In a proof-of-concept test, both excellent specificity and sensitivity were achieved. This platform could detect target DNA down to 5 fM and exhibited strong robustness. More interestingly, this technique showed an impressive fast detection process. The current widely used RT-qPCR technique usually requires 1–3 h to complete. By contrast, this CQDs-based DNA detection method could be completed within 5 min. Similarly, a novel dual-mode optical platforms based on sulfur-doped CQDs was reported for fluorescence detection and Raman spectroscopy analysis of virus was reported, with a detection limit down to 0.1 fg mL^−1^ [221].

Developing lateral flow immunoassay for COVID-19 detection is regarded as a reliable alternative method for RT-qPCR test. Compared with RT-qPCR test, lateral flow immunoassay can give the test result within 5–15 min and without the need of complicated machine improving the detection efficiency [222]. Xu et al*.* reported a lateral flow assay that utilizes carbon dots/SiO_2_ nanospheres (CSNs) as tracer for qualitative and quantitative evaluation of severe fever with thrombocytopenia syndrome virus (SFTSV) [223]. The design of the lateral flow strip in their work is similar to a typical colloidal gold-based lateral flow test strip while the chromogenic reagents were replaced from gold nanoparticles to silanized CDs. The strips were consisted of a sample pad, a nitrocellulose membrane, an absorbent pad and a black plastic adhesive card. The test line and control line were functionalized with anti-SFTSV monoclonal antibody and goat anti-mouse IgG antibody, separately. Upon the loading of CSNs and virus samples to the sample pad, the mixture of virus and CSNs migrated to the absorbent pad under capillary action. Since the CSNs were pre-coupled with anti-SFTSV monoclonal antibody, the virus and CSNs would be captured on the test line forming a sandwich structure while the excess CSNs would be captured on control line. Under ultraviolet light, by analyzing fluorescence signal, qualitative results and semi quantitative results could be obtained. Compared with traditional immuno-gold lateral flow strip, this CDs-based assay showed a 2 orders of magnitude lower detection limit at 10 pg mL^−1^. Furthermore, the silane treatment of CDs effectively prevented the fluorescence reduction and quenching caused by CDs’ aggregation.

Very recently, Li et al*.* successfully developed a practical method that utilized CQDs to detect SARS-CoV-2 [224]. This method was based on the magnetic relaxation switches (MRSw) effect of magnetic CQDs in nuclear magnetic resonance (NMR) analysis. The magnetic CQDs were synthesized by doping Gd^3+^ in CQDs. After that, specific antibody, which is targeting to spike (S) protein of SARS-CoV-2, was coated to the surface of magnetic CQDs. The switch of relaxation was generated upon the binding between magnetic CQDs and virus. The concentration of virus was determined based on the relaxation change. As a result, this method can detect SARS-CoV-2 as low as 248 particles mL^−1^. This method has the advantages of high specificity, time saving, low cost and low contamination risk. The average detection time and detection cost of this method were 2 min and $1.25. Also, since the sample tube was completely sealed during the entire testing process, the risk of viral leakage was effectively minimized.

According to the World Health Organization’s report, airborne transmission is the main mode of COVID-19 contagion [225]. In the present scenario, developing antiviral personal prospective equipment (PPE) may help to fight the COVID-19 pandemic. Raghav et al*.* presented a perspective on whether graphene-based materials can be virucidal [226]. Due to the distinct electroconductive properties, graphene and graphene-derivatives can interact with biomacromolecule. Using graphene-derivatives as fabric or mist spray can thus block the entry and/or contact of virus [227]. In addition, introducing graphene-derivatives to positively charged filters in air purification or air-conditioning devices would likely facilitate the filtration of virus in environment.

With respect to the antiviral treatment of COVID-19, CQDs could have a great potential [228]. In a recent report, Alizadeh et al*.* systemically reviewed and analyzed the efficacy of nanoscale materials against coronaviruses [229]. They screened the publications which focused on antiviral study of nanomaterials between 1945 and 2020 and identified 21 studies after some meta-analyses. It was found that there was a positive relationship between the efficacy of nanoscale materials and coronaviruses in vitro and in animal models. More interestingly, the particle size of nanomaterials showed little effect on antiviral ability while the shape of nanomaterials had great impact on antiviral effect. The spherical nano particles further had stronger antiviral ability, which was about 39% higher than other types of nano-morphologies in the studies of Middle East respiratory syndrome–related coronavirus (MERS-CoV). By taking account of the average particle size and shape of CQDs, these nanomaterials could offer some positive effects against coronaviruses.

In another work, Łoczechin used small molecular precursor, citric acid and ethylenediamine, to synthesize CQDs via hydrothermal process [19]. The prepared CQDs were then coupled with different boronic acid containing compounds resulting in boronic acid modified CQDs. Compared with non-modified CQDs, the introduction of boronic acid led to the inactivation of human coronavirus 229E (HCoV-229E), with an estimated EC_50_ at 52 ± 8 μgmL^−1^, while the addition of mannose however resulted in a loss of the inhibition effect. The mechanism behind this phenomenon could be attributed to the boronic acid groups which can interact with glycan units on the surface of virus and form tetravalent complexes selectively and reversibly. Hence, in this study boronic acid modified CQDs acted as pseudo lectins which conjugated to the envelope glycoprotein S of coronavirus, leading to the inactivation of virus. Further research showed that different modification modes resulted in different antiviral activities. For example, doping boron acid groups to CQDs could improve their antiviral activity against human coronavirus with an enhanced EC_50_ value of 5.2 ± 0.7 μgmL^−1^.

In order to combat the COVID-19 pandemic, the global vaccine research and development have been boosted while maintaining the highest standards on safety. At present, the CQDs-based vaccine delivery platform and CQDs-based adjuvant strategy have gained more attentions and have being verified. Li et al*.* developed a CQDs-based intranasal vaccine delivery platform to induce specific immune response [230]. The researchers synthesized CQDs by using chitosan and branched polyethyleneimine as raw materials through microwave-assisted pyrolysis. After that, negatively charged antigen such as ovalbumin was attached to CQDs via electrostatic interaction. The capture and internalization of antigen/CQDs composite by dendritic cells was observed under confocal laser scanning microscope while in the control group where CQDs were not contained, no antigen was internalized by cells at 2 and 6 h. Furthermore, it was found that CQDs-based vaccine formulations could retain at the mucosal sites for a long time with stronger across-mucosa antigen transportation, promoting antigen absorption and presentation. Compared with bare antigen, mice which were vaccinated with antigen/CQDs composite exhibited antigen-specific immune response and induced more memory T cells. Such a high immune boosting ability could be attributed to the permeation enhancement effect and promoted antigen transport. In another study, Cheng et al*.* evaluated the application potential of CQDs as vaccine adjuvant [231]. The majority of chickens which were treated with vaccine and CQDs exhibited antiviral immunity after the second immunization. Compared with traditional Freund’s adjuvant, CQDs showed stronger immune efficacy in this study.

## Conclusions and perspective

Since CQDs were first reported in 2004, they have attracted increasing attentions due to their excellent intrinsic properties (e.g. electronic, optical, mechanical, chemical and thermal properties). These superior nanomaterials could be synthesized easily and fast by top-down and bottom-up approaches using various carbon sources. They have already been successfully applied in many biomedical fields, such as biosensing, bioimaging, drug and gene delivery, photothermal therapy, and recently in virus detection and inhibition and treatment of viral infection. In the recent decade, great progress has been made in the development of CQDs, including synthetic method, purification process, modification/functionalization approach and property tuning. However, some remaining challenges are yet to be conquered. For example, it is still difficult to assemble some CQDs with high efficiency and quality, and to predict the reproducibility of their optical and physical properties. The better understanding of relationship between surface passivation and photoluminescence is still required.

CQDs have successfully been applied as functional nanomaterials in optical and electrochemical sensing of virus and they could further displace or enhance the function of traditional detective probes in bioassays for virus detection. Amid the unprecedented pandemic of COVID-19, CQDs have demonstrated some promising capabilities to facilitate the detection process of various pathogenic viruses including SARS-CoV-2 with excellent sensitivity and accuracy.

They have also been prosperously exploited and applied as antiviral agents for the inhibition and/or disinfection of pathogenic viruses, by interfering virus entry and uncoating, boosting immune response, and interfering virus biosynthesis. More interestingly, they have shown a broad spectrum of antiviral activity against some life-threatening flaviviruses and non-enveloped viruses.

Along with the future development and better understanding of CQDs, more effective and greener preparation methods (e.g. microwave-based method) could be developed using low-cost and nature carbon sources, resulting in scale-upped production with less batch differentia.

## Data Availability

Not applicable.

## Abbreviations

ACE2: Angiotensin converting enzyme 2
AlNPs: Aluminum nanoparticles
APCs: Antigen-presenting cells
AuNPs: Gold nanoparticles
BQAS: Biquaternary ammonium salt
CDs: Carbon dots
CdTe QDs: Cadmium telluride quantum dots
COVID-19: Coronavirus disease 2019
CPDs: Carbonized polymer dots
CQDs: Carbon quantum dots
CSNs: Carbon dots/SiO_2_ nanospheres
CT: Computerized tomography
Cur-CQDs: Curcumin-based carbon quantum dots
DRF: Damage response framework
ELISA: Enzyme-linked immunosorbent assay
FTIR: Fourier-transform infrared spectroscopy
GQDs: Graphene quantum dots
HAART: Highly active anti-retroviral therapy
HAV: Hepatitis A virus
HBV: Hepatitis B virus
HCoV-229E: Human coronavirus 229E
HEV: Hepatitis E virus
HIV: Human immunodeficiency virus
HSV-1: Herpes simplex virus type 1
IFN: Interferon
KSHV: Kaposi’s sarcoma-associated herpesvirus
LNA: Locked nucleic acid
MGPs: Magnetic glass particles
MRSw: Magnetic relaxation switches
MP-MoO_3_ QDs: Magnetic-derivatized plasmonic molybdenum trioxide quantum dots
MERS: Middle east respiratory syndrome
MERS-CoV: Middle east respiratory syndrome–related coronavirus
MTX: Mitoxantrone
MWCNT: Multi-wall carbon nanotube
NAAT: Nucleic acid amplification test amplification test
NMR: Nuclear magnetic resonance
NNRTI: Nucleoside reverse transcriptase inhibitors
NPs: Nanoparticles
NRTI: Non-nucleoside reverse transcriptase inhibitors
OVA: Ovalbumin
PCR: Polymerase chain reaction
PPE: Personal prospective equipment
PRV: Pseudorabies virus
PRRSV: Porcine reproductive and respiratory syndrome virus
QDs: Quantum dots
QY: Quantum yield
ROS: Reactive oxygen species
RTB: Ricin toxin binding subunit B
RT-PCR: Real-time polymerase chain reaction
RT-qPCR: Reverse transcriptase quantitative polymerase chain reaction
SARS: Severe acute respiratory syndrome
SARS-CoV-2: Severe acute respiratory syndrome coronavirus 2
SFTSV: Severe fever with thrombocytopenia syndrome virus
S-gCNQDs: Sulfur-doped graphitic carbon nitride quantum dots
SiNPs: Silica nanoparticles
SPR: Surface plasmon resonance
TEM: Transmission electron microscope
XRD: X-ray diffraction
